# Hybrid Deep Learning Model for Date Palm Disease Classification: A Fusion of HybridConv Mixer and Vision Transformer

**DOI:** 10.1002/fsn3.71086

**Published:** 2025-10-21

**Authors:** Taifa Ayoub Mir, Salil Bharany, Rupesh Gupta, Rania M. Ghoniem, Ateeq Ur Rehman, Belayneh Matebie Taye

**Affiliations:** ^1^ Chitkara University Institute of Engineering and Technology Chitkara University Rajpura Punjab India; ^2^ Department of Information Technology, College of Computer and Information Sciences Princess Nourah bint Abdulrahman University Riyadh Saudi Arabia; ^3^ School of Computing Gachon University Seongnam‐si Republic of Korea; ^4^ Department of Computer Science, College of Informatics University of Gondar Gondar Ethiopia

**Keywords:** agricultural automation, automated disease detection, data augmentation, date palm disease detection, early disease, ensemble models, hybrid convolutional mixer, vision transformer

## Abstract

Date palms sustain agricultural operations in dry areas, yet they encounter two serious diseases: brown spots and white scale, leading to harvest degradation and inferior production. The current practice of manual detection shows both inefficient processing along with substantial human error, thus requiring automated disease classification systems. The proposed research develops a disease identification system for date palms by merging the capabilities of Hybrid Convolutional Mixer (HybridConv) and Vision Transformer (ViT). The HybridConv Mixer focuses on detecting local disease patterns alongside ViT, enhancing global feature analysis, thus resulting in better disease classification. The trained model operated on brown spots and white scale, and healthy date palm leaf images from a dataset that received data augmentation for increased model reliability. The ensemble model demonstrates outstanding performance by reaching 99.89% accuracy, which outperforms single Convolutional Neural Networks (CNN) models in terms of precision, recall, and F1‐score, thus providing promising technology for date palm cultivation disease detection automation.

## Graphical abstract

The study proposes a hybrid deep learning model combining HybridConv Mixer and Vision Transformer (ViT) to classify date palm leaf diseases—specifically brown spots and white scale—with high accuracy. The ensemble model achieved 99% classification accuracy, outperforming individual models. This fusion approach enhances both local and global feature extraction, offering a reliable and efficient solution for automated agricultural disease detection.

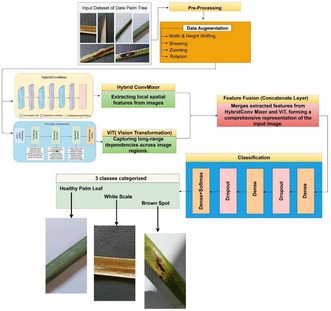

## 1 Introduction

The date palm species *Phoenix dactylifera* L. remains among the most ancient cultivated fruit‐bearing plants, which grow throughout extensive areas of the Middle East and North African regions alongside South Asian regions. Many countries depend on date palms for economic and agricultural success because they enhance their food security, along with their commercial trade activities. The hot, dry climate conditions that date palms thrive in make them an excellent sustainable food source for areas where the water supply is limited. Millions of people worldwide depend on date fruit as their staple food because it contains carbohydrates, vitamins, minerals, dietary fiber, and other essential nutrients. Dates hold great value in nutrition and prolonged shelf stability, which leads to their widespread use in fresh consumption and the production of processed food for date syrups, confectionery, and nutritional supplements industries.

The cultivation of date palms provides economic and dietary value through farming methods that create employment opportunities across the spectrum of farming activities and export industries that process and sell dates. Interest in dates worldwide is growing, with Saudi Arabia, Egypt, Iran, and Algeria dominating the leading market segment. Date palm cultivation faces considerable challenges due to the spread of numerous plant diseases throughout production areas, resulting in reduced yields, poor product quality, and general crop deterioration. Rapid identification of these diseases, followed by proper intervention, proves essential for high farming productivity alongside continuous farming sustainability (Djemai et al. 2021).

Several diseases distinctively affect date palm plants, resulting in significant economic consequences. The fungi, along with bacteria and insects, cause diseases that damage photosynthesis functions and inhibit the plant's natural fruiting ability. The types of pathogens determine the range of symptoms, where pathogen‐inflicted palms can show either minor damage or total leaf loss. Diseases spread quickly during humid periods, so it is crucial to detect them early to prevent widespread destruction. Both farmers and agricultural experts traditionally use manual visual methods to detect diseases, but these approaches show limitations because they are subjective, require much time, and can cause false diagnoses.

The combination of computer vision with deep learning techniques provides a new foundation for automated plant disease diagnosis, offering improved speed and accuracy, as well as enhanced scalability. Deep learning models use image‐based classification to specify and distinguish multiple plant diseases through functioning without significant human operator involvement. The research aims to build an advanced deep learning system designed for identifying date palm illness types, including brown spots and white scale disease.

### 1.1 Brown Spot

The fungal pathogen Graphiola phoenicis causes brown spots, which are also known as Graphiola leaf spot. Symptoms from this disease develop into small brown to black circular lesions, which expand and result in necrotic tissue formation on the leaves. The fungal pathogen disperses its spores through the wind or water molecules while also spreading using contaminated equipment. Brown spots that are left unattended lead to severe damage, reducing photosynthetic processes and causing weakness in plant growth, which in turn results in reduced fruit yield. Commercial date palm plantations are significantly affected by the brown spots disease, as infected trees produce lower yields with deteriorated fruit quality.

Spraying fungicides during the early stages of brown spot infection proves efficient in controlling these symptoms. Excessive use of fungicides creates environmental problems, while plants might develop resistance against these chemicals. Effective control of brown spots depends on early intervention through automated disease classification models because manual identification methods fall short as an adequate control measure (Bengio et al. 2013).

### 1.2 White Scale

White scale disease damages date palms as its causing agent is the Parlatoria blanchardi insect, which creates white waxy deposits on palm leaves. White waxy manifestations appear on leaves because the insect larvae dwell within the deposits. The continuous spread of infestation results in leaves that turn yellow and become thin and fragile until they cannot properly take in nutrients. Unattended trees develop major leaf problems, which decrease their fruit yield and eventually lead to total tree death.

The detection of white scale disease depends on time‐sensitive identification to permit effective use of conventional control measures based on insecticides and biological agents. Severe infestations occur when detection is delayed, necessitating additional pesticide applications that cause environmental damage and promote insect resistance to pesticides. Metallicina.eu utilizes automated deep learning‐based image classification as an efficient detection method for white scale disease that enables precise pest management approaches without environmental harm (Bilal et al. 2025).

### 1.3 Healthy

Date palm leaves maintain a regular green appearance because they lack any signs of lesions, color changes, or shape abnormalities. The correct identification of healthy versus diseased leaves remains essential for deep learning model training to detect infections at different intensities. The classification systems enable precision agriculture to develop, as farmers can utilize AI‐based plant health monitoring to reduce unnecessary pesticide use and enhance disease control (Chollet 2017).

Plant disease classification has become more accurate with the emergence of deep learning techniques as a new approach. Plant disease detection and classification, benefiting extensively from applications of Convolutional Neural Networks (CNNs), achieve high accuracy levels. The spatial features extraction capability of CNNs enables the model to identify how diseases manifest through image patterns. Plant disease detection algorithms commonly employ several deep learning architectures such as ResNet, MobileNet, and EfficientNet, which have achieved promising evaluations according to research findings (Kaur, Mittal, et al. 2025).

Conventional CNN architectures struggle to handle the diverse symptoms of diseases that appear differently under various environmental conditions, including lighting changes and background disturbances. Vision Transformers (ViTs) have been integrated by researchers to address the obstacles within agricultural disease classification systems. The global analysis approach of ViTs surpasses CNNs' local interest because these transformers provide a superior ability to identify structural plant leaf defects.

The research presents a Hybrid Deep Learning Model consisting of both Hybrid Convolutional Mixer (HybridConv) and ViT components for better disease classification results. The HybridConv Mixer effectively retrieves spatial features in lower levels while the ViT component excels at identifying distant image relationships. Disease classification accuracy and robustness improve significantly when combining local and global feature extraction methods.

The training dataset, derived from Kaggle's “Date Palm Data,” comprises 2631 images categorized into three types: Brown Spots (470 examples), Healthy (1203), and White Scale (958). The model generalization receives an enhancement through data augmentation procedures, which incorporate rotation and scaling with shearing and image flipping. The data distribution includes training and validation sections that amount to 75% and 25%, respectively, to achieve both balanced representation and better model learning.

Main contributions from this work consist of:

1. A HybridConv Mixer model unites convolutinary layers with batch normalization features to simultaneously extract shallow and deep properties, resulting in enhanced accuracy for date palm disease classification.
2. The implementation of ViT as a backbone enables the model to analyze image patches by capturing their long‐range dependencies, thereby detecting complex patterns in date palm images. This leads to enhanced detection accuracy.
3. An ensemble system integrating HybridConv Mixer and ViT models generates better features and classifications from datasets. The ensemble technique combines different models to achieve higher performance in date palm disease classification by exploiting their beneficial features.

## 2 Related Work

Table 1 presents a comparative analysis of recent studies on date palm classification and disease detection, including the models used, accuracy achieved, limitations, and future scope.

**TABLE 1:** Related work overview on date palm disease and quality detection by the techniques of machine learning and deep learning.

References	Focus	Techniques used	Best accuracy	Limitations	Future suggestions
Rybacki et al. (2024)	CNN model for classifying date palm fruit varieties	CNN, Data Augmentation (rotation, zooming, flipping)	93%	Lighting sensitivity, occlusion	Attention mechanisms, multi‐spectral imaging
Namoun et al. (2024)	Dataset and classification of infected vs. healthy leaves	ResNet50, EfficientNet, MobileNetV2	~95% (EfficientNet)	Class imbalance	GANs, thermal + hyperspectral imaging
Hessane et al. (2023b)	Stage‐wise classification of white‐scale disease	Pretrained CNNs, ResNet50	94.50%	Class imbalance	GANs, AI‐IoT integration
Alaa et al. (2020)	Machine learning for palm disease detection	SVM, RF, KNN, Color Segmentation, Texture Analysis	~91% (SVM)	Lighting variation, handcrafted features	Edge detection, DL fusion with hyperspectral
Alshehhi et al. (2022)	Leaf discoloration detection via transfer learning	VGG16, ResNet50, DenseNet	96% (DenseNet)	Data scarcity	Ensemble learning, multispectral imaging
Abu‐zanona et al. (2022)	Palm disease detection using CNN	Custom CNN, ResNet, InceptionNet	94.20%	Mobile deployment	MobileNetV2

The research examines how CNNs can classify various date palm fruit varieties. Traditional classification methods struggle to identify different date palm fruits due to their similar texture patterns and matching color schemes. The authors established their own CNN model for high‐definition fruit imaging after implementing data augmentation methods using rotation, zooming, and flipping operations. The model achieved 93% accuracy, demonstrating its success in categorizing fine‐grained samples. The method faced difficulties when dealing with changes in lighting and areas that were obstructed from view. The authors propose better accuracy through the combination of attention‐based mechanisms with multi‐spectral imaging. The study develops an AI‐controlled date palm quality evaluation system that supports agricultural and commercial markets (Rybacki et al. 2024).

The analysis presents infected and healthy date palm leaf data for enhancing deep learning systems that diagnose diseases. The research collects high‐definition images that show various stages of white scale infection and brown spots, as well as sudden decline syndrome. The benchmark tests conducted with ResNet50, EfficientNet, and MobileNetV2 revealed that EfficientNet achieved the highest accuracy, reaching ~95%. The research places significant value on dataset balance while recommending GANs for synthetic image creation to enhance the distribution of classes. The proposal entails the combination of thermal and hyperspectral imaging technology to detect diseases during their earliest stages (Namoun et al. 2024).

A research investigation evaluates various deep learning models that have been pretrained to identify white‐scale disease stages in date palms. A two‐stage disease categorization process detects disease occurrence initially, followed by a mechanism to ascertain its advanced stage. Among the tested model variations, ResNet50 demonstrated a peak accuracy level of 94.5%. The research recognizes a problem with class imbalances during disease classification and proposes GAN‐based synthetic augmentation as a solution for enhanced performance. The study investigates the combination of AI technology with IoT monitoring systems for live disease identification. The proposed system helps precision agriculture operations by identifying diseases early and enhancing crop health maintenance (Hessane et al. 2023b).

The presented work integrates image processing methods with machine learning strategies to detect palm tree diseases. The technique performs color segmentation, texture analysis, and feature extraction before using SVM, Random Forest, and KNN for classification. The artificial intelligence technique known as SVM delivered the highest accuracy rate of approximately 91%, thus proving its effectiveness as a texture‐based classifier. These methods depend heavily on labor‐created features; therefore, they experience limitations in different lighting situations and environmental effects. The research established that deep learning models, particularly CNNs, help improve classification performance by their ability to identify essential features automatically. Disease detection performance might be enhanced through further examination of edge detection techniques and hyperspectral imaging, along with deep learning fusion methods in future research (Alaa et al. 2020).

Researchers have used deep transfer learning to identify leaf discoloration because it serves as a primary warning sign of date palm diseases. A custom dataset containing discolored and healthy leaves was submitted to VGG16 and ResNet50 along with DenseNet for their fine‐tuning process. The highest accuracy rate of 96% was obtained using DenseNet, but VGG16 presented the optimal balance of accuracy with efficient computation. Transfer learning methods reduce the training period and are particularly effective in agricultural artificial intelligence applications, where systems often lack sufficient labeled examples. Ensemble learning with multiple models, along with multispectral imaging detection, serves to enhance disease recognition performance (Alshehhi et al. 2022).

The research investigates the classification of date palm diseases using a CNN architecture, creating a specialized CNN model and comparing it against ResNet and InceptionNet. The proposed CNN model operates on different palm diseases with 94.2% accuracy while demonstrating better performance than traditional machine learning methods. The authors advise MobileNetV2 as the best option for mobile disease detection due to its combined low resource usage and computational speed (Abu‐zanona et al. 2022).

The researchers establish a novel K‐Means clustering and deep learning algorithm system for automatic Sudden Decline Syndrome (SDS) detection in date palm trees. The method separates leaf areas through K‐Means clustering so the deep CNN model can perform classification processes. The developed model demonstrates a 94.17% successful distinction between SDS‐damaged leaves along with healthy leaves. The research highlights the need for better tools to identify features, along with a proposed integration of multiband imaging technology for improved detection accuracy (Magsi et al. 2023).

Deep learning analysis and GIS‐based remote sensing are used to identify Dubas bug infestations in date palm trees through this research. The authors utilized drone imagery to train their model, which is based on YOLO, and achieved 95% accuracy. The study demonstrates that deep learning systems gain the capability to detect infestations during their early stages, which makes it possible for the drone‐based automatic pest surveillance system (Al‐Mulla et al. 2023).

The research project depends on Unmanned Aerial Vehicles (UAV) imagery to perform large‐scale date palm tree classification. The deep CNN model processed high‐resolution aerial images and reached an accuracy level of 96.3%. The study proposes that palm cultivation decision support will improve through deep learning integration with GIS‐based spatial analytical tools (Gibril et al. 2021).

The research introduces a prediction model for date palm mite infestation through artificial intelligence approaches. The researchers created a deep learning hybrid system using CNN features and decision trees, which reached 95% accuracy in predictions according to the research (Mohammed et al. 2023).

The research work presents DeepPalm as a deep learning‐based model that identifies various date palm types. Traditional methods of classification encounter difficulties in achieving high precision when differentiating various date palm species because of their similar morphological attributes. DeepPalm adopts CNN‐based feature extraction, which detects subtle textural, shaped, and colored differences between diverse date palm groups. The model reached ~94% accuracy levels, surpassing those of conventional machine learning methods. Data augmentation procedures enable the authors to improve model generalization while battling bias. The DeepPalm model represents a significant development toward automatic palm categorization systems that benefit both farmers and agricultural researchers, as well as industrial users in their date palm species identification tasks (Ali et al. 2024).

This research develops DPXception, a minimally complex CNN especially designed for both efficient and precise date palm species classification. Xception architecture serves as a base for the model through its implementation of depthwise separable convolutions that achieve high performance at reduced computational complexity. Research introduces measures to enhance model operational efficiency, enabling it to run effectively on edge and mobile devices, which supports real‐time agricultural applications. DPXception achieves 95% accuracy on a large dataset of date palm images for differentiating between different species. The research presents evidence of minimal model dimensions and response duration while describing DPXception as a strong technology platform for field‐based identification systems. The next steps in research should combine either transformer‐based models or multi‐spectral imaging methods to boost classification resistance (Safran et al. 2024).

The investigation centers on UAV‐based remote sensing together with multispectral imaging to detect diseases in palm groves. The researchers use high‐resolution aerial drone images to detect early signs of disease in date palm trees. Using deep learning models trained with images obtained from UAVs allows the study to achieve strong disease detection accuracy for white scale, along with brown spots and sudden decline syndrome. The research findings demonstrate that UAV platforms with multispectral camera systems produce valuable information about plant health, which allows early detection systems for widespread farms. The study shows how UAV‐based precision agriculture systems will decrease human disease monitoring requirements and improve farming through smart management operations (Casas et al. 2023).

The research offers a specially prepared dataset of date palm leaves with diseases that help deep learning scientists build effective disease recognition systems. The analysis includes high‐quality labeled images that show Dubas insect, white scale disease, and fungal infection symptoms on leaf structures. A detailed annotation process of datasets helps CNN‐based disease detection models achieve better accuracy rates, according to the authors. Experiments using EfficientNet provided the best results to classify diseases with an accuracy of approximately 95%, while MobileNet and ResNet50 also provided significant performance. This research highlights the challenges associated with class imbalance, suggesting that GAN‐based synthetic data generation offers a viable solution. The dataset functions as an essential standard for upcoming research that allows AI‐driven precision agriculture to enhance early‐stage disease identification (Al‐Mahmood et al. 2023).

This study in (Kaur, Al‐Yarimi, et al. 2025) and Shandilya et al. (2025) presents a hybrid deep learning model for plant disease classification by combining CNN (EfficientNetB3) and Vision Transformer‐like architecture (InceptionResNetV2) with genetic algorithm optimization. It ensures high accuracy and balanced performance using SMOTE for class imbalance and advanced data augmentation. Explainable AI tools like SHAP and LIME are integrated to enhance model transparency. The approach is adaptable for real‐time, edge‐based diagnosis of date palm leaf diseases.

The research design introduces Deep‐PDSC as a deep learning‐based system that detects different stages of Parlatoria Date Scale (PDS) outbreaks in palm trees. Date palms develop severe crop damage because the PDS pest infests plant leaves, stems, and fruits when early detection does not occur. The research introduces an infected leaf image classification system using CNN deep learning to detect PDS stages at various disease levels. The analysis demonstrates that ResNet50 delivers optimal performance at 93.5% accuracy out of the examined pretrained CNN architectures, which also include VGG16 and Xception. A solution to address early‐stage infection data imbalance requires data augmentation approaches, according to the researchers' findings. The research supports merging IoT real‐time monitoring systems and hyperspectral imaging technologies to detect PDS at its early stages, which enables farmers to take prompt protective measures against severe infections (Hessane et al. 2023a).

The research aims to develop artificial intelligence methods that detect male and female date palm trees at early developmental stages. Plantation efficiency and overall fruit output depend on early gender identification among date palm trees because these plants display dioecious characteristics. The identification of male and female date palm trees through traditional methods requires both genetic testing and waiting until flowering occurs, but this process is time‐consuming and costly. The authors developed a machine learning predictive model that employs leaf morphology data and spectral readings to identify gender and sex from young date palm seedlings. The experimental design combines Random Forest and SVM, along with CNN, until the CNN reaches an accuracy level of approximately 91%. Research indicates that combining hyperspectral imaging with deep neural networks (DNNs) will further enhance gender classification precision. The study results are crucial for precision farming operations, enabling farmers to optimize land use, reduce costs, and improve plantation efficiency through the early identification of male and female trees (Naeem et al. 2023). It has shown that sophisticated architectures of deep learning are effective in detecting crop diseases. The proposed AI‐based MetaFormer architecture is an olive leaf disease, which was suggested by Pacal et al. (2025) and could perform efficiently and autonomously in a complicated agricultural setting. Likewise, Pacal and Işık (2025) mixed CNNs with ViTs to detect the diseases of the leaves on the corn plant more accurately, which also testifies to the benefits of the hybrid structure in the classification task. Continuing on this topic, a recent study (Pacal 2024) utilized a massive dataset of maize leaves with a state‐of‐the‐art Vision Transformer model and showed that transformer‐based architecture can boost crop yield and sustainability by providing powerful disease detection.

## 3 Proposed Methodology

**FIGURE 1:**
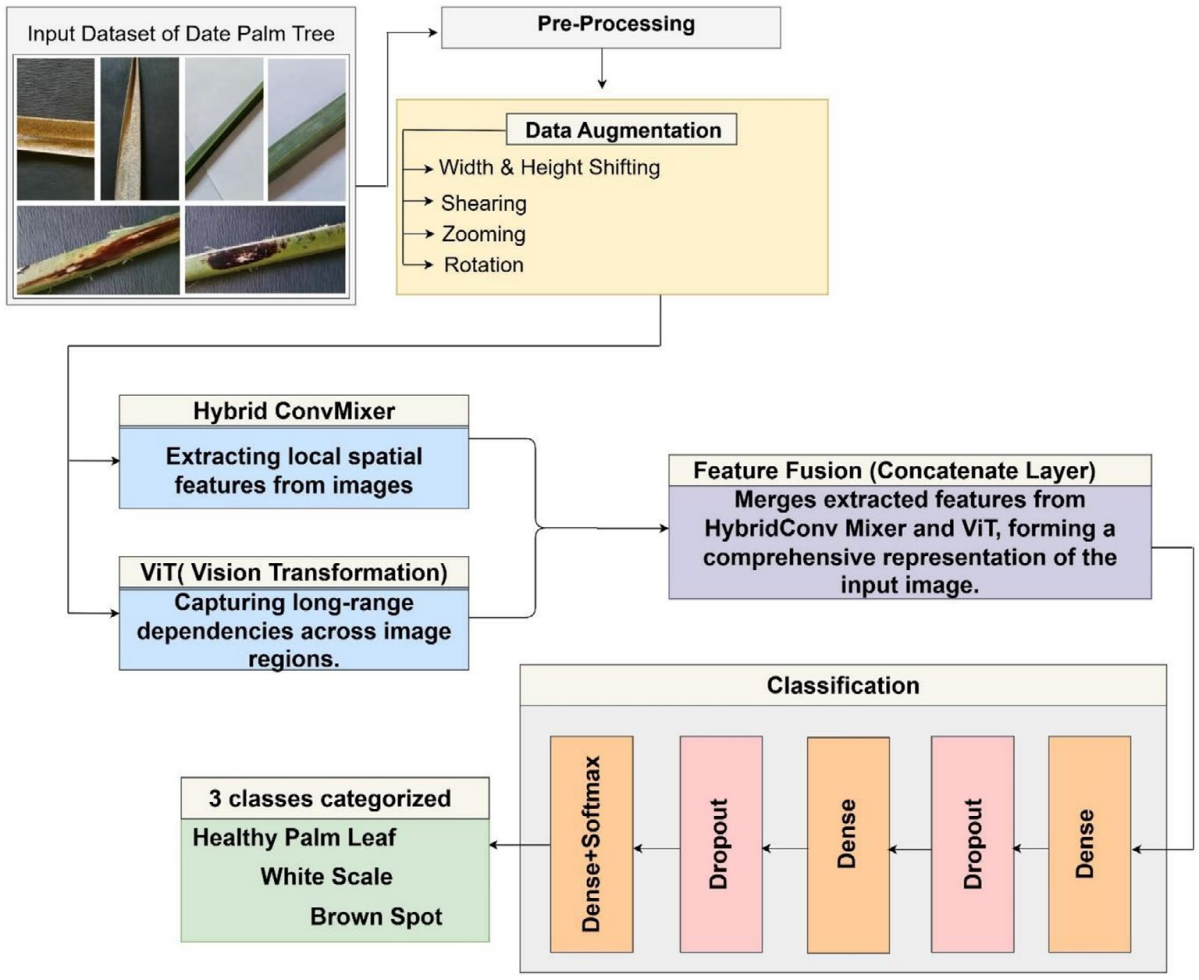
Proposed methodology.

In Figure 1, the proposed study utilizes a hybrid deep learning framework that combines HybridConv and ViT to automate date palm disease classification. HybridConv extracts the local features of leaf texture, edge, and lesion patterns, while ViT searches for relationships across image regions. A combined output of the networks gets compiled into a unified feature representation that serves for classification purposes.

The solution has a sequential workflow starting with data processing, then moving to augmentation steps, followed by HybridConv and ViT feature extraction, after which prediction occurs through ensembling. Through this methodology, the model utilizes both localized and global features to enhance its accuracy rate and resistance to environmental condition changes.

### 3.1 Dataset Description

This study used two publicly available datasets to test the performance and generalization ability of the proposed model: the Date Palm Data by Kaggle, which has 2631 labeled images of three classes (Brown Spots, Healthy, and White Scale) and the Infected Date Palm Leaves by Dubas Insects dataset which has 3000 drone‐captured images of four classes (Healthy, Infected only, Infected only, and Infected mixed).

#### 3.1.1 Input Dataset 1

The Kaggle data set (Hamaidi 2025) obtained in the first dataset has 2631 labeled images belonging to three different categories: Brown Spots (470 images): palm leaves that have brown lesions that show fungal infection, Healthy (1203 images): healthy leaves with no symptoms of the disease, White Scale (958 images): white scale disease is a disease that attacks leaves, which are covered with white waxy parasites due to bugs.

**FIGURE 2:**
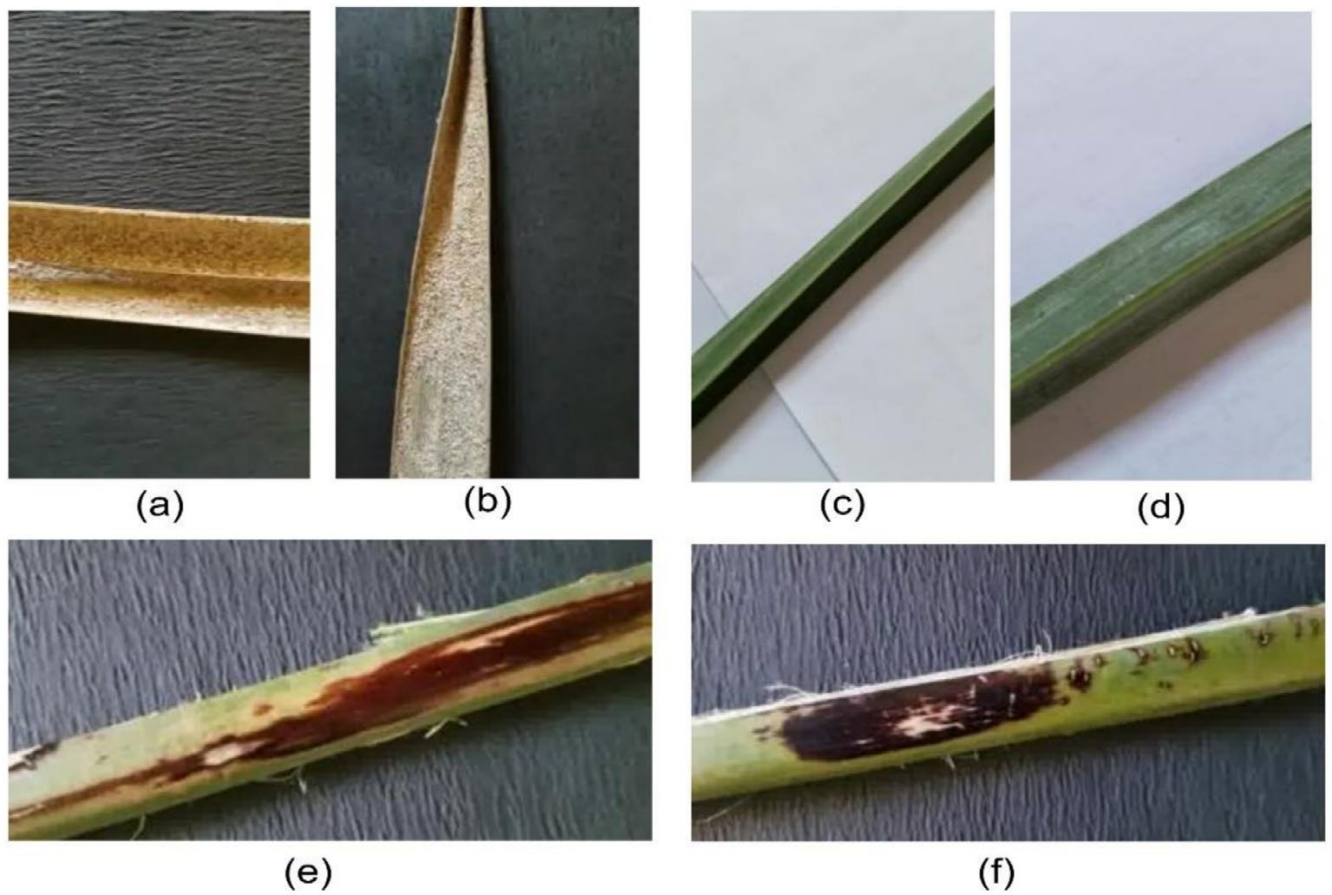
Data set images of date palm tree (a) white scale, (b) white scale, (c) healthy date leaf, (d) healthy date leaf, (e) brown spot, (f) brown spot (Hamaidi 2025).

As depicted in Figure 2, the dataset has images of date palm leaves with different levels of development and health conditions, which is adequate to train and test models of disease classification.

#### 3.1.2 Input Dataset 2

**FIGURE 3:**
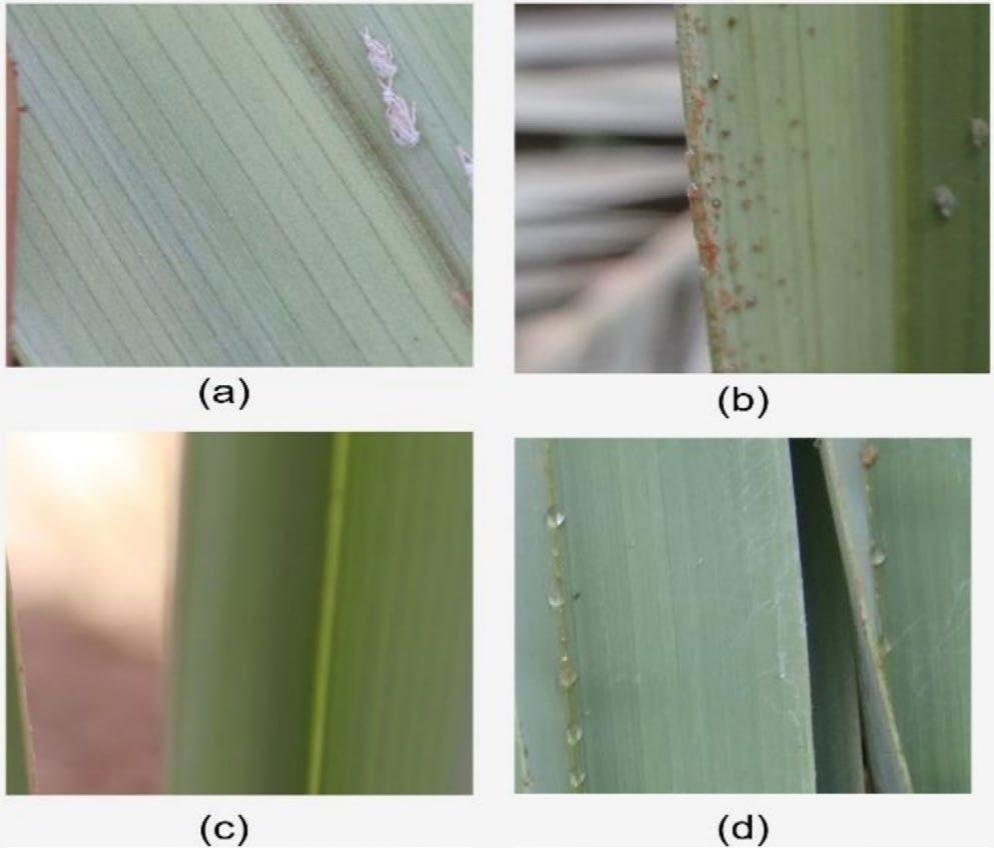
Dataset on infected date palm leaves by dubas insects (a) infected bug (b) infected dubas (c) healthy date leaf (d) infected honey (Magsi et al. 2023).

The second dataset (Magsi et al. 2023) is Infected Date Palm Leaves by Dubas Insect that comprises 3000 photographs of the field conditions of the drone cameras usage. The palm leaf images are classified into four: Healthy (800 images): leaves with no infection symptoms, Infected—Bugs only (600 images): the leaves with Dubas insects of various life stages, the third generation nymphs up to adults, Infected—Honeydew alone (800 images): leaves of honeydew secretions produced by the insects, Infected—Mixed (Bugs + Honeydew, 800 images): leaves with both insects and honeydew shown in Figure 3.

This dataset would be especially useful in comparing the level of infestation, estimating the population of insects, and identifying the degree of damage in the environment of actual agricultural conditions. The combination of both datasets, which dealt with the symptoms of fungal and scale diseases, and the second one, which dealt with the insect‐generated infestations, allowed us to test the proposed model in a wide range of disease cases, acquisition routes, and environments and, thus, make it more robust and applicable to the real world.

The images received a sizing operation that set each one to 224 × 224 pixels to achieve standardization for every sample while maintaining deep learning system compatibility. The data is divided into 75% training data and 25% dedicated for validation and testing purposes to maintain balanced learning conditions. The distribution follows in Equation (1):

(1) $$D=\left\{\left(x_{i},y_{i}\right)\right\}$$

where $x_{i}$ represents image sample, $y_{i}$ represents corresponding class label.

The dataset is splitted into training (75%) and validation/testing (25%) shown in Equations ((2), (3), (4)).

(2) $$\text{Trainning}\;\mathrm{set}:D_{\text{train}}\subset D\;\text{with}\;\left|D_{\text{train}}\right|$$

(3) $$\text{Validation}\;\mathrm{set}:D_{\mathrm{val}}\subset D\;\text{with}\;\left|D_{\mathrm{val}}\right|$$

(4) $$\text{Testing}\;\mathrm{set}:D_{\text{test}}\subset D\;\text{with}\;\left|D_{\text{test}}\right|$$

The three‐way split hereby ensures that model training is performed separately from Cross Validation (for hypertuning and early stopping) and testing (for performance evaluation and confusion matrix generation only).

### 3.2 Preprocessing

Deep learning models require preprocessing of image data to optimize consistency and achieve better generalization for their machine learning process. The HybridConv Mixer + ViT model requires several preprocessing operations to convert the image input into a common data format. Model robustness depends heavily on data augmentation and normalization techniques because they help the model adapt successfully to various real‐life image variations.

#### 3.2.1 Data Augmentation

The training process of deep learning models heavily depends on a significant amount of data for achieving generalization capability. Small‐scale datasets of plant diseases usually present difficulties in acquisition for research purposes. The necessity for data augmentation techniques emerges because they generate artificial variations of training images to increase their diversity. These techniques include:

1. Rotation ($\theta \sim u\left(-30^{{}^{\circ}},30^{{}^{\circ}}\right)$): the model generates different leaf orientation views through random image rotations.
2. Width and height shifting ($∆w,∆h\sim u\left(-0.2,0.2\right)$): accounts for variations in camera positioning.
3. Shearing ($\sigma \sim u\left(-0.2,0.2\right)$): introduces the distortions in image perspective.
4. Zooming ($\gamma \sim u\left(0.8,1.2\right)$): enhances model robustness by adjusting image scale.
5. Horizontal flipping: randomly flips the images, which introduces variability in leaf positioning.

**FIGURE 4:**
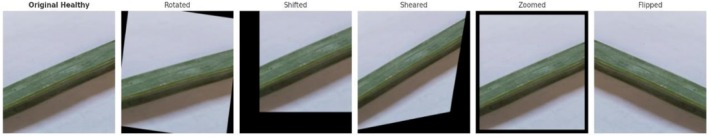
Data augmentation on healthy leaf images.

The data augmentation process for healthy date palm leaves is visually explained through Figure 4 by showing transformations that boost model generalization capabilities. The baseline image maintains its position, but the rotated version performs simulation through random angle adjustments between −30° and 30°. Camera position variations are included in the shifted image through both horizontal and vertical translation. Different views of features become recognizable through image shearing since it distorts the image perspective. Model learning functions with adjusted feature detection abilities as the zoomed image changes its scale between 0.8× and 1.2×. Identifying leaf structures with greater robustness is promoted through the implementation of flipping, which adds mirrored image variations. The augmentations work synergistically to make the model perform better in reality through expanded dataset diversity, along with decreased overfitting while improving classification accuracy.

**FIGURE 5:**
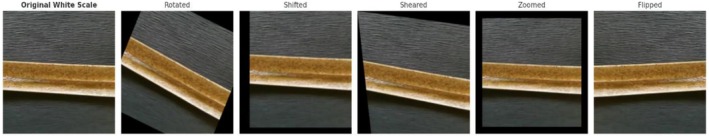
Data augmentation on white scale disease images.

The illustration in Figure 5 displays multiple transformations of a date palm leaf image affected by white scale disease to improve model robustness. The original format shows the untreated picture, yet the rotational transformations within +30° and –30° help the system detect damage regardless of image direction. In the simulated images, camera movement effects are represented using horizontal or vertical translation of the image content. Shearing functions change image perspective so models become better prepared to handle distortions in image architecture. The zoomed version controls the modified scale levels from 0.8× to 1.2× to help the model recognize elements that appear at different distances. Multiplying the image horizontally allows the dataset to achieve more diversity through mirrored image variations. Augmented features in the model enhance its ability to identify white scale disease under various lighting and angle conditions and at different scale levels, which decreases overfitting and improves practical outcomes.

**FIGURE 6:**
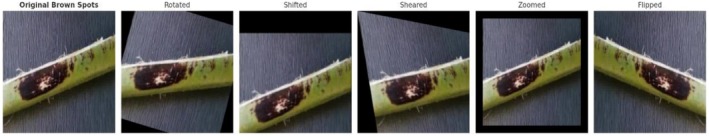
Data augmentation on brown spots disease images.

The image presentation utilizes data augmentation on a date palm leaf affected by brown spots through different modifications for improved model generalization performance, as shown in Figure 6. The original image maintains its original state, and the rotational version adjusts from ±30° to assist the model with spotting brown spots across different orientations. The shifted image modifies the dimensions through horizontal and vertical translations because of camera position modifiers. Sheared images modify their visual representation to enhance the model's capability when detecting structural changes in images. This transformation enlarges or shrinks the image dimensions from 0.8 times to 1.2 times to educate the model about diseases at varied distances. Horizontal image mirroring extends the dataset by adding mirrored reflection variations. Other built‐in features enhance the model's ability to recognize brown spots in various situations, which in turn decreases overfitting and improves recognition accuracy.

The initial data set was class imbalanced, with 470 Brown Spots, 1203 Healthy, and 958 White Scale leaves. In response, we only performed purposive data augmentation on the training set (75% of the dataset) and left the validation and the test set untouched in order to provide a fair assessment. Operations such as augmentation like rotations (+30 and −30°), zoom, horizontal flipping, shearing, and translations were performed randomly. Under this strategy, the minority population (Brown Spots) was increased fourfold to 1760 training samples, whereas Healthy and White Scale were increased to about 1900 training samples each, which created a more balanced training distribution as presented in Table 2. This balancing enhanced the learning representative features in all classes by the model, and fair evaluation was obtained on the original unaugmented test set.

**TABLE 2:** Class distribution before and after augmentation (training set only).

Class	Original images	Before augmentation training set (75%)	After augmentation (training only)	Validation/test (25%, no augmentation)
Brown Spots	470	352	1760	118
Healthy	1203	902	1900	301
White Scale	958	718	1900	240
Total	2631	1972	5560	659

### 3.3 Proposed Ensemble Model

**FIGURE 7:**
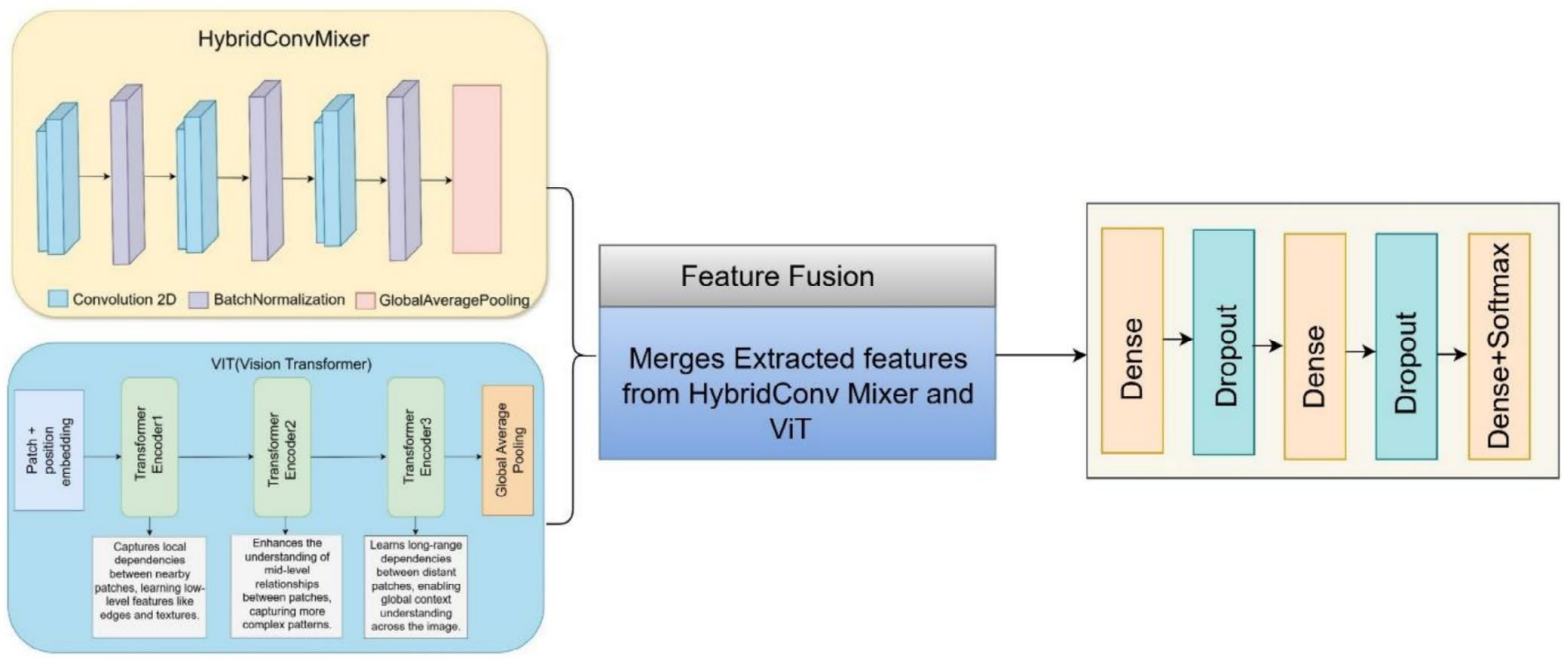
Proposed ensemble model.

The illustration in Figure 7 shows a proposed ensemble model architecture that combines the feature extraction approaches of HybridConv and ViT. The HybridConv Mixer performs convolutional operations that use subsequent batch normalization, then global average pooling to obtain hierarchical features from image inputs. The extracted features from HybridConv are then fed into the ViT, where operators perform image patch processing and apparatus position embeddings. Subsequently, the encoders facilitate local, mid‐level, and long‐range connections throughout the image. Both models transmit their extracted features to the feature fusion block for unification. The new block integrates HybridConv Mixer features with ViT features to provide the model with improved capabilities for analyzing low‐level and high‐level image structures. The model incorporates fully connected layers (Dense) and dropout layers for preventing overfitting after the feature fusion step. The model delivers the prediction outcomes by using a softmax layer, which produces class label results. Combining the approaches of HybridConv Mixer and ViT through an ensemble method produces better results for image classification tasks.

#### 3.3.1 Convolutional Mixer (ConvMixer)

**FIGURE 8:**
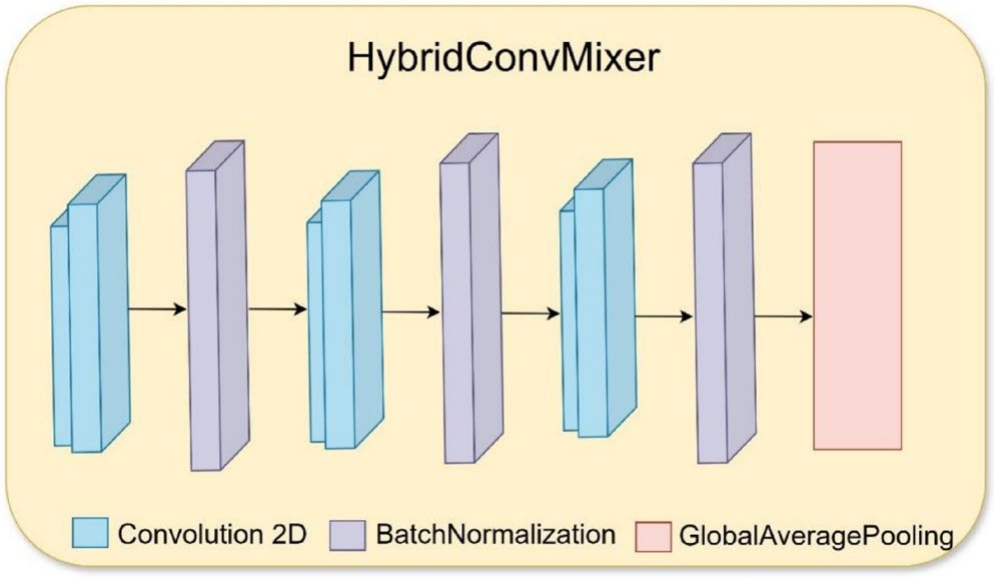
Architecture of the HybridConv Mixer model.

The subsequent operation following preprocessing focuses on feature extraction, where important image patterns are derived to help with classification tasks. Through the HybridConv Mixer stage, the model extracts detailed spatial features which help it differentiate between normal and diseased date palm leaves. The model uses three convolutional layers with Batch Normalization enabled after each one to maintain training stability, while ReLU activation introduces non‐linearity into the process shown in Figure 8. The first convolutional layer detects simple visual factors, including edges, textures, and minor differences in leaf color and shape patterns. The data processing through successive network layers enables the model to identify disease‐specific elements together with lesions and leaf discolorations. Feature distribution stability results from Batch Normalization, and this technique prevents internal covariate shifts, together with ReLU activation, enabling faster training because it cancels negative activations.

Through a hierarchical framework, the model develops its image content understanding by successively refining its knowledge base. The HybridConv Mixer produces a condensed high‐dimensional feature representation during its final layer before the ViT module uses it to learn classification accuracy through global context.

The convolutional operation takes the following form, shown in Equation (5):

(5) $$f_{l}=\sigma \left(W_{l}*x+b_{l}\right)$$

where $f_{l}$ represents the feature map at a layer, $W_{l}$ is the convolutional kernel, * denotes the convolution operation, $b_{l}$ is the bias term, σ is the activation function (ReLU).

After extraction, the proposed model implements GAP on the extracted features to transform the maps into a 128‐dimensional vector, as shown in Equation (6).

(6) $$F_{\text{Conv}}=\mathrm{GAP}\left(f_{l}\right)$$

Table 3 represents the HybridConv Mixer architecture, which is responsible for extracting spatial features from date palm leaf images. The first Conv2D layer applies 32 filters of size (3,3), detecting basic edge patterns with 896 trainable parameters. A BatchNormalization layer follows, stabilizing activations and accelerating convergence with 128 parameters. The second Conv2D layer increases filters to 64, allowing the network to learn more complex textures, contributing 18,496 parameters. Another BatchNormalization layer with 256 parameters ensures feature normalization. The third Conv2D layer expands filters to 128, extracting fine‐grained spatial details, using 73,856 parameters. The final BatchNormalization layer contains 512 parameters, maintaining stability before applying GlobalAveragePooling2D, which reduces dimensionality by averaging each feature map into a 128‐dimensional vector. This step compacts the extracted spatial features for further processing while preserving essential information, ensuring efficient feature representation before concatenation with ViT‐based global features.

**TABLE 3:** Layer configuration of the HybridConv Mixer model.

Layer name	Layer type	Output shape	Number of parameters
conv2d	Conv2D	(224, 224, 32)	896
batch_normalization	BatchNormalization	(224, 224, 32)	128
conv2d_1	Conv2D	(224, 224, 64)	18,496
batch_normalization_1	BatchNormalization	(224, 224, 64)	256
conv2d_2	Conv2D	(224, 224, 128)	73,856
batch_normalization_2	BatchNormalization	(224, 224, 128)	512
global_average_pooling2d	GlobalAveragePooling2D	(128)	0

#### 3.3.2 Vision Transformer (ViT) Backbone

The Vision Transformer operates as a global extractor that learns image relationships through self‐attention computations between various image areas. ViTs process data by analyzing overall images with self‐attention, while CNNs operate through local field connections.

**FIGURE 9:**
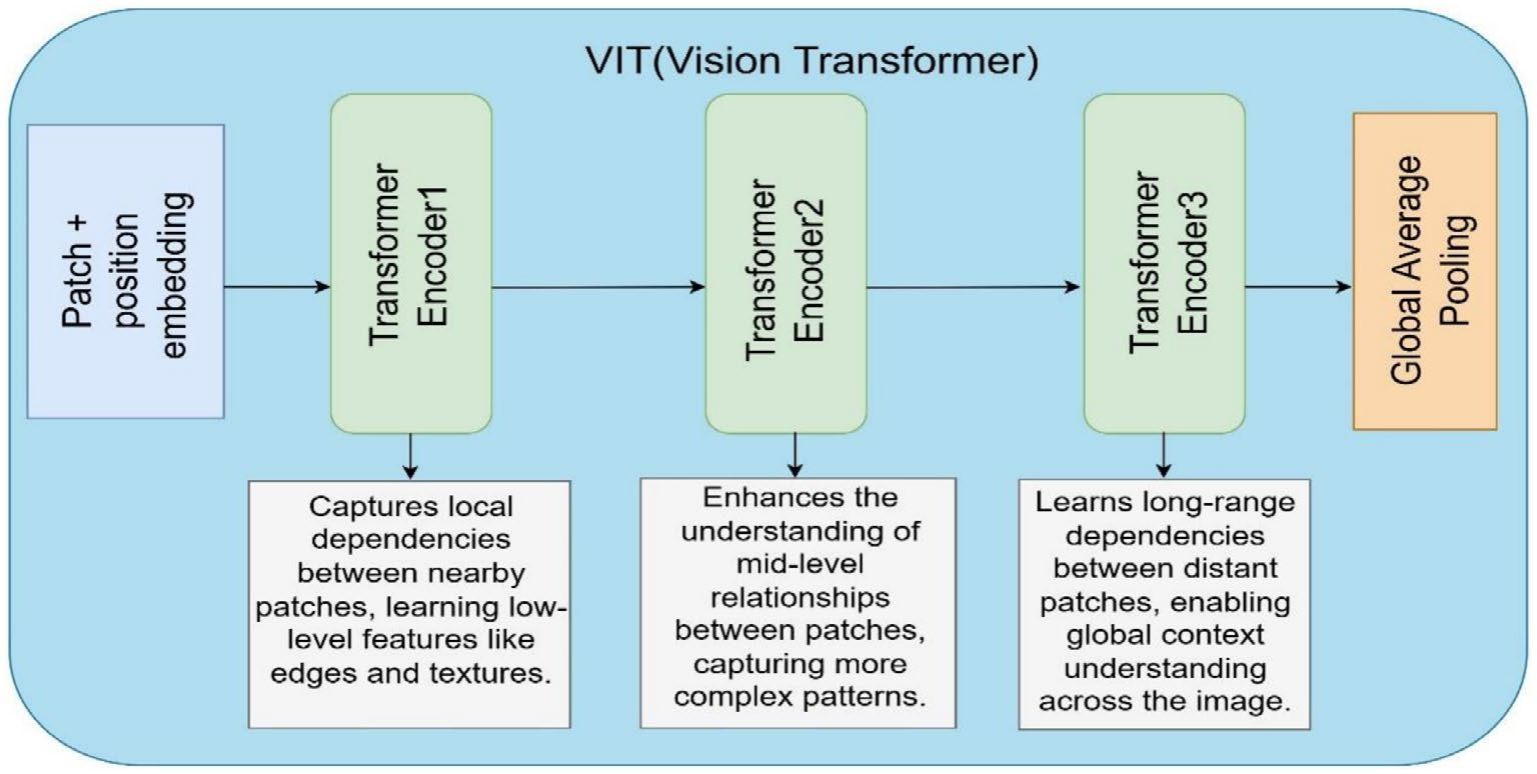
Architecture of the ViT model.

The beginning of the ViT architecture, as shown in Figure 9, applies position embeddings to the divided image patches obtained from the input image. The system sends the divided patches to several Transformer Encoder blocks. The first Transformer Encoder functions to detect neighborhood relationships between adjacent image sections that identify edges and textures as fundamental features. The second Transformer Encoder emphasizes the identification of mid‐level patch relations, which enables the model to discover more advanced patterns. The third Transformer Encoder adds extended capability by teaching the model to detect dependencies between both nearby and distant patches, which leads to a whole‐image contextual comprehension. Global Average Pooling consolidates the acquired features into a brief representation usable for classification and other operational needs. The architecture applies self‐attention functionality, which produces spatial structure at multiple abstraction levels.

All images undergo non‐overlapping patching before each segment is projected linearly into a D‐dimensional space, as shown in Equation (7).

(7) $$Z_{0}=\left[P_{1}W_{E;}P_{2}W_{E};\ldots ;P_{N}W_{E}\right]+E_{\mathrm{pos}}$$

where $P_{1}$ is a patch, $W_{E}$ is the embedding matrix, $E_{\mathrm{pos}}$ represents positional encodings to retain spatial information.

The self‐attention mechanism is computed as:

(8) $$Z_{l+1}=\text{Softmax}\;\left(\frac{QK^{T}}{\sqrt{\mathrm{dk}}}\right)\;V$$

where Q, K, and V are the Query, Key, and Value matrices derived from the embedded patches shown in Equation (8).

#### 3.3.3 Ensembling and Fusion Process

The model applies an ensemble approach that combines features produced by HybridConv with features from ViT to access both local and global perspectives. The HybridConv Mixer generates output as $F_{\text{Conv}}$ which consists of 128 features obtained from three convolutional layers alongside Batch Normalization and Global Average Pooling. The $F_{\text{Conv}}$ The vector includes detailed spatial information containing essential leaf disease symptom features, such as visible texture and local features, which aid diagnosis. The ViT component, as described earlier, generates an output global feature vector $F_{\mathrm{vit}}$ which has 1280 dimensions. The two related interpretations merge into this sequence shown in Equation (9), resulting in a 1408‐dimensional hybrid feature vector.

(9) $$F_{\text{fusion}}=\text{Concatenate}\;\left(\left[F_{\text{conv}},F_{\mathrm{vit}}\right]\right)$$

The acquisition of feature‐level concatenation, as opposed to prediction‐level methods that present fusion at the level of predictions, which included averaging or majority vote, was prompted by the fact that this preserves the representational power of both local and global feature spaces. With this approach, coarse fusion can be done at the early stage, which leaves additional fully connected layers to learn complex and non‐linear interactions between the spatial texture and contextual semantics. In contrast to weighted averaging or attention‐based approaches that require additional resources, such as processing power and model tuning, concatenation is relatively straightforward, computationally expedient, and experimentally successful. The design creates an effective, no‐dilute fusion of complementary features along with maximum information with minimum interference that leads to a good performance in classification.

The Concatenate() layer accomplishes this operation. After vector combination, the system flows into two series of fully connected layers containing 256 neurons and 128 neurons, with dropout operation for overfitting prevention. The final softmax layer outputs class probabilities for the three disease categories (“Brown Spots,” “Healthy,” “White Scale”). The combination of local convolutional and global transformer‐based feature extraction in this ensembling process leads to a robust, accurate classifier system for date palm diseases.

Table 4 presents the integration of the HybridConv Mixer and ViT that forms the final layers of the hybrid deep learning model for date palm disease classification. Hence, the first layer, named Concatenate, unifies feature representations between the two models into a single vector containing 1408 dimensions to serve as an input for classification. Subsequently, the Dense layers transform these refined features for use in the decision process. The first Dense layer transforms extracted features by applying non‐linear transformations, enabling it to learn sophisticated patterns between features using 256 neurons and 360,704 parameters. The subsequent Dropout layer contains 256 neurons but introduces no new learnable parameters because it uses random neuron deactivation to stop overfitting during the training phase. The Dense layer, with 128 neurons and 32,896 parameters, provides an additional refinement to the representation, followed by another Dropout layer with 128 neurons and no parameters to maintain generalization strength. The output Dense layer (3 neurons, 387 parameters) performs a softmax activation to determine class probabilities for the healthy, brown spot, and white scale disease categories, enabling the model to select correct classifications.

**TABLE 4:** Fully connected layers and softmax classification for HybridConv Mixer + ViT model.

Layer name	Layer type	Output shape	Number of parameters
concatenate	Concatenate	(1408)	08
dense	Dense	(256)	360,704
dropout	Dropout	(256)	0
dense_1	Dense	(128)	32,896
dropout_1	Dropout	(128)	0
dense_2 (Softmax)	Dense + Softmax	(3)	387

## 4 Results and Discussion

To enhance the performance of the model, the Adam optimizer was used with a fixed learning rate of 0.0001. Adam was chosen because it supports adaptive learning and has been demonstrated to be stable concerning a range of deep learning tasks, in the combination of convolutional and transformer elements. Training on a batch size of 32 provided a trade‐off between computational efficiency and convergence speed and was appropriate in the model training.

The primary hyperparameters of training used to train the deep learning models in this paper are depicted in Table 5. The Adam optimizer was chosen because it has an adaptive learning rate, which generates faster convergence and improves generalization in deep architectures. During the training, the learning rate of 0.0001 was held constant to ensure stability. The batch size of 32 was chosen for the model's training, taking into account both memory considerations and training speed. As the loss function, categorical crossentropy was selected because it is appropriate to use when classification is multiclass or when there is a need to classify brown spots, healthy leaves, and white scale disease in date palms. All these decisions resulted in effective training and the reproductive performance of the experiment.

**TABLE 5:** Summary of key training hyperparameters.

Parameter	Value	Description
Optimizer	Adam	Adaptive optimizer for stable and efficient learning
Learning rate	0.0001	Fixed learning rate used during training
Batch size	32	Number of samples processed per batch
Loss function	Categorical cross entropy	Suitable for multi‐class classification

### 4.1 HybridCONV MIXER Model Performance Metrics: Epoch‐By‐Epoch Accuracy and Loss

The epoch‐wise accuracy and loss values for the HybridConv Mixer model are shown in Table 6. The table showcases crucial learning progressions of the model across multiple epochs, providing detailed information on training accuracy and loss, as well as validation accuracy and loss at various steps. At epoch 1, the model exhibits low predictive capability, with a training loss of 1.2736 and a validation loss of 0.8222. The model demonstrates fast learning of meaningful patterns from data since training accuracy hit 88.53% and validation accuracy rose to 89% during epoch 5. Training accuracy at epoch 10 reached 95.14%, while validation accuracy reached 91.59%, and the model exhibited declining loss rates for both training and validation. The classifier demonstrates strong performance at epoch 20 because it achieves 97.53% training accuracy and 94.14% validation accuracy. The learning phase nears optimal at epoch 25 because training accuracy reached 98.29% while validation accuracy maintained 94.23% performance. During early stopping at epoch 27, the training loss achieves a minimum of 0.0556, but validation accuracy reaches its peak of 94.52%, suggesting that training should stop. The HybridConv Mixer model demonstrates excellent performance for classification accuracy and consistently stable validation results. The model performs well in extracting classification features from data across various epochs, demonstrating its effectiveness in real disease detection tasks.

**TABLE 6:** Epoch vise accuracy and loss for HybridConv Mixer.

Epoch	Train loss	Train accuracy	Val loss	Val accuracy
1\100	1.2736	0.5794	0.8222	0.7357
5\100	0.3751	0.8853	0.3696	0.89
10\100	0.1768	0.9514	0.2933	0.9159
15\100	0.1143	0.9693	0.241	0.9318
20\100	0.085	0.9753	0.2121	0.9414
25\100	0.0623	0.9829	0.2204	0.9423
27\100 (early stop)	0.0556	0.9865	0.2301	0.9452

**FIGURE 10:**
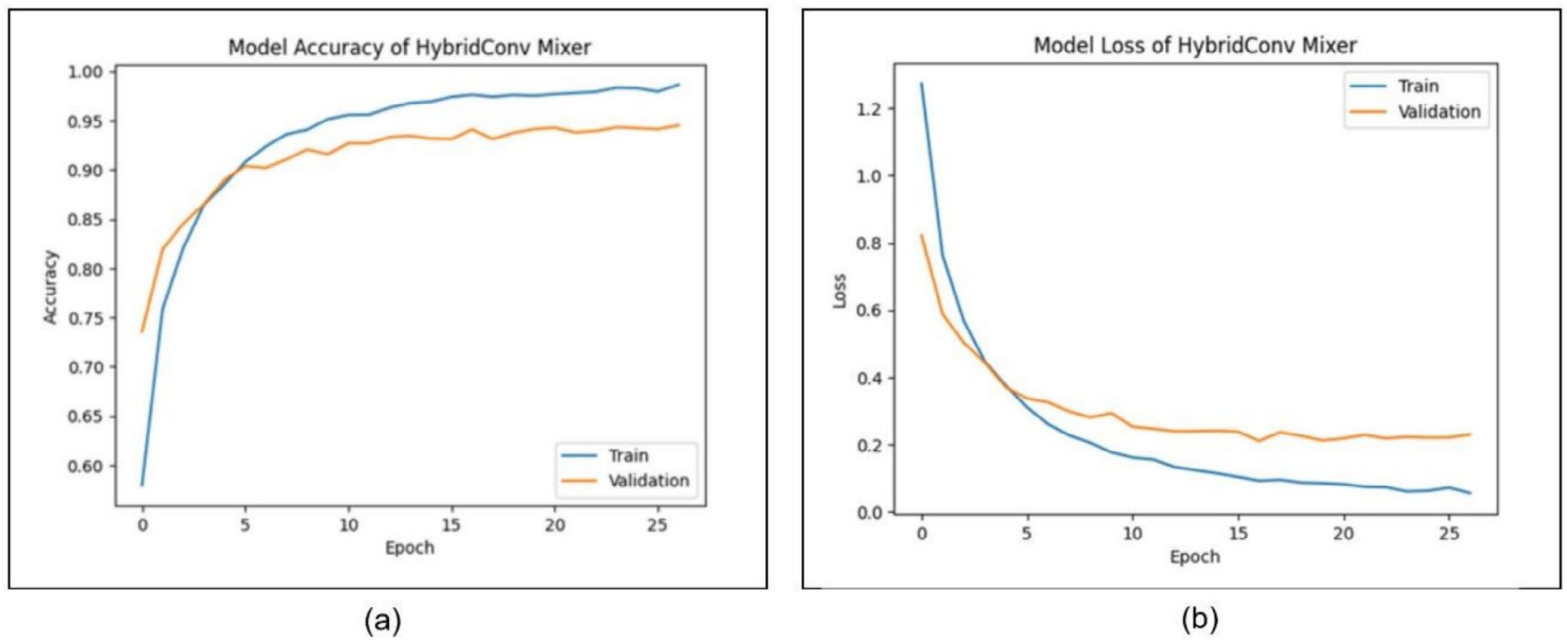
Training and validation results for HybridConv Mixer: (a) accuracy, and (b) loss.

Figure 10 shows the performance of the HybridConv Mixer model during training and validation for 25 epochs. In subfigure (a), the trends of the accuracy during training are displayed, whereas subfigure (b) represents the trends of the loss during training. Moreover, in Figure 10a, training and validation accuracy are rising with time. At the final stage of training, the accuracy reaches close to 0.99, whereas the validation accuracy finds a more stable 0.95. The curves are reasonably proximate with one another, indicating no significant difference between the training and validation stages in the model. Taken as a whole, this shows that the model can learn the features well and generalizes to unseen data with minimal deviation. Figure 10b also indicates that training loss is lower in the initial epochs and gradually becomes lower till the late rounds. The validation loss is trending the same, with a slight plateau after the 10th epoch, and the loss is leveling off at about 0.2. Although the validation loss yields a higher loss on average compared to the training loss, the difference is not significant, indicating minimal overfitting. In general, the loss and the accuracy diagrams show that the HybridConv Mixer model has a stable training outcome. The learning curves demonstrate the model's capability to achieve an acceptable performance in the specified classification task and its generalization ability to validation data over the training epochs.

#### 4.1.1 Evaluation of Validation/Testing Results

**FIGURE 11:**
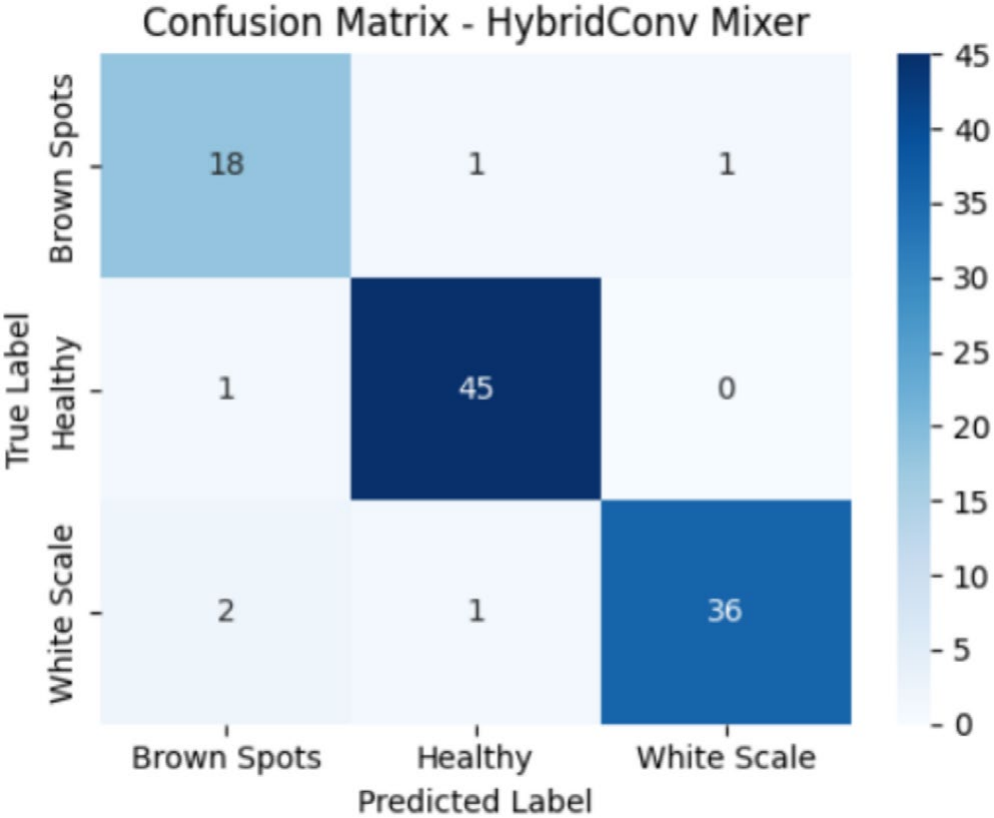
Confusion matrix for HybridConv Mixer model on test data.

Figure 11 represents the confusion matrix of the HybridConv Mixer model, which answers the question of its performance on the test set. The model has achieved an accuracy of 18 out of 20 images, correctly classifying them as Brown Spots, with one misclassified as Healthy and another as White Scale. In the case of Healthy leaves, 45 out of 46 samples were correctly predicted, and one was incorrectly classified as Brown Spots. Under White Scale, 36 of the 39 samples were identified correctly, two were misidentified as Brown Spots, and one was identified as Healthy. The whole model has a reasonably good performance, especially when detecting Healthy leaves. There is, nevertheless, a certain degree of confusion between Brown Spots and White Scale, possibly as the result of some cases of each having similar visual symptoms. The confusion matrix indicates that, although the model performs well in most cases, there is a need to improve the distinction between specific disease categories, particularly where signs and symptoms overlap.

Table 7 presents the classification report of the HybridConv Mixer model, evaluating its ability to predict the three sample classes: Brown Spots, Healthy, and White Scale. In the case of the Brown Spots, the model had a precision of 0.86 with a recall of 0.90 and an F1‐score of 0.88, indicating some misclassification in predicting this class. The healthy class achieved a higher performance, with a precision of 0.96, a recall of 0.98, and an F1‐score of 0.97, indicating that the model performs well at detecting healthy samples. In the case of the White Scale class, the model achieved a pretty good result, recording 0.97 precision, 0.92 recall, and 0.95 F1‐score with a small portion of missed cases. The macro average based on the three classes was 0.93 in precision, recall, and the F1‐score, implying consistent performance. The model's accuracy level was 94%, indicating that most predictions for all classes in the test set were accurate.

**TABLE 7:** Classification report for hybrid Conv mixer.

Class	Precision	Recall	F1‐score	Support	Accuracy
Brown spots	0.86	0.9	0.88	20	0.99
Healthy	0.96	0.98	0.97	46	
White scale	0.97	0.92	0.95	39	
Macro avg	0.93	0.93	0.93	105	

### 4.2 Vision Transformation (ViT) Model Performance Metrics: Epoch‐By‐Epoch Accuracy and Loss

The ViT model demonstrates its training development through epoch‐wise accuracy and loss measurements, which are presented in Table 7. The training and validation accuracy numbers increase across multiple epochs and decrease simultaneously as the model gains better classification skills. At epoch one, the model begins with training loss at 1.1426 while the validation loss stands at 0.7544 before achieving training accuracy of 62% alongside validation accuracy of 79.71%. The model demonstrates early learning behavior because it continues to produce incorrect predictions at this stage. The model demonstrates fast feature learning from the dataset at epoch 5, as both accuracy rates increased substantially to 88.06% for training and 90.10% for validation, while loss values decreased substantially. Training accuracy achieves 92.90% at epoch 10 while validation accuracy reaches 92.60% at the same time point, as the loss values continue to decrease. At epoch 20, the model reaches 96.27% training accuracy while validating at 95.19%, showing excellent skills in both classification and feature extraction. Training accuracy reached 96.39% while validation accuracy became 95.38% at epoch 25 as the loss continued decreasing. The model reaches its highest level of performance during epoch 30 since validation accuracy maintains steady marks at 95.10% while training accuracy achieves 97.22%. The model reached early stopping at epoch 36 with training accuracy at 97.10% while validating accuracy stabilized at 95.29%, indicating no additional training value. The ViT model effectively learned from the dataset through the achievement of high classification accuracy along with consistent validation performance (Table 8).

**TABLE 8:** Epoch wise accuracy and loss for ViT.

Epoch	Train loss	Train accuracy	Val loss	Val accuracy
1\100	1.1426	0.6200	0.7544	0.7971
5\100	0.3679	0.8806	0.3023	0.9010
10\100	0.2140	0.9290	0.2268	0.9260
15\100	0.1383	0.9511	0.2072	0.9394
20\100	0.1126	0.9627	0.1784	0.9519
25\100	0.1026	0.9639	0.1708	0.9538
30\100	0.0858	0.9722	0.1984	0.9510
36\100 (early stop)	0.0820	0.9710	0.2134	0.9529

**FIGURE 12:**
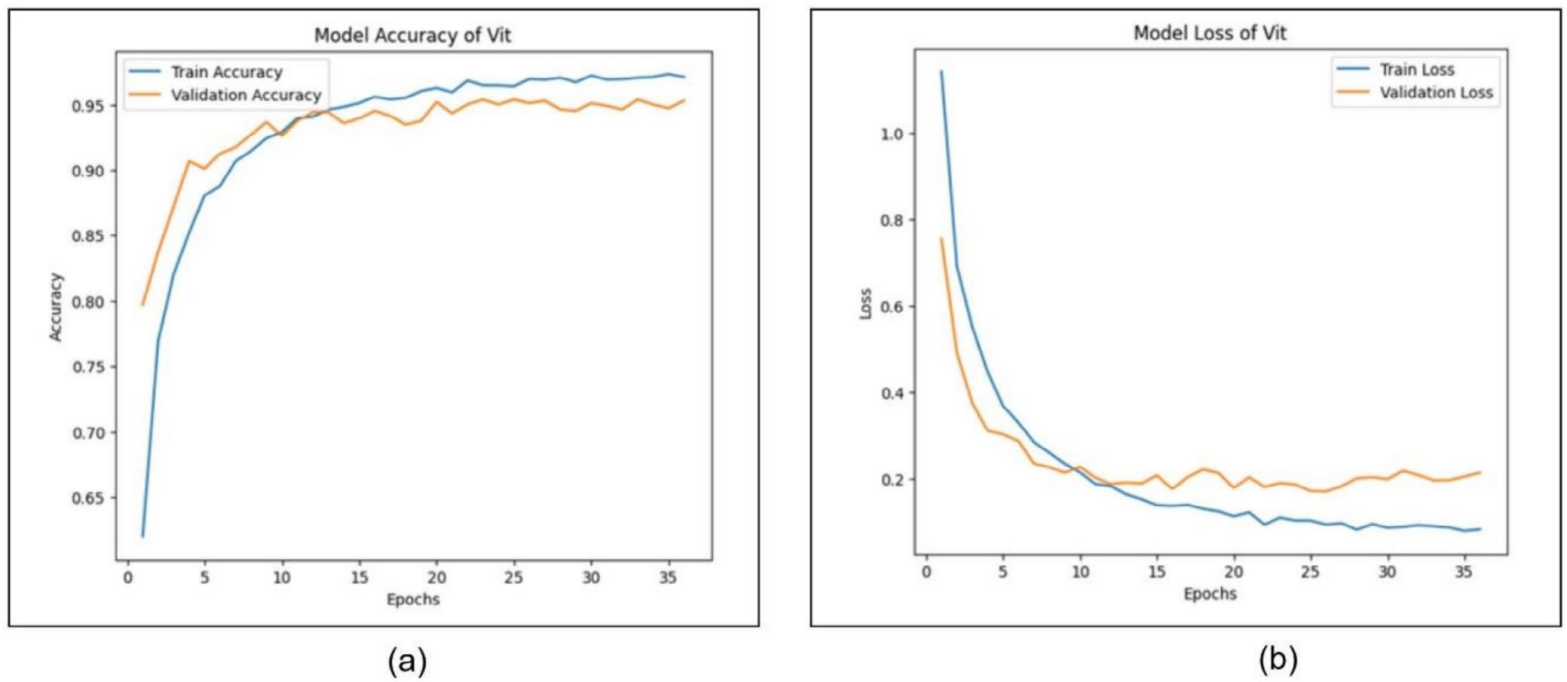
Training and validation results for vision transformation (a) accuracy and (b) loss.

The training and validation accuracy of the ViT model, which happens on 35 epochs, is shown in Figure 12. The accuracy of the model in terms of training and validation is displayed in subfigure (a), and the loss during the same training and validation is shown in subfigure (b). The training precision of the ViT model is also rising steadily, as depicted in Figure 12a, and taking a value of a little over 0.96 at the end of training. The accuracy of validation also exhibits a similar trend, reaching close to 0.95. Such a tight convergence of training and validation accuracy curves implies that the ViT model fits the training data to a great extent but with very weak indicators of overfitting. The sudden increase in accuracy in the first epochs indicates an effective learning rate and ability to quickly extract features, which are characteristic of the transformer‐based architecture. Figure 12b shows the loss curves of training and validation. The two losses start falling sharply in the initial few epochs, illustrating that the model is minimizing error well. The loss on the train decreases over time, whereas the loss on validate stops dropping below epoch 20 and remains at a low value. Finally, the analyses shown in Figure 12 once again prove the efficiency of the algorithm, that is, the fact that the ViT is very accurate and that its performance is not affected by any serious underfitting or overfitting. The fact that it ensures a tight training and validation convergence increases its appropriateness for the investigation of the plant disease classification task.

#### 4.2.1 Evaluation of Testing Results

**FIGURE 13:**
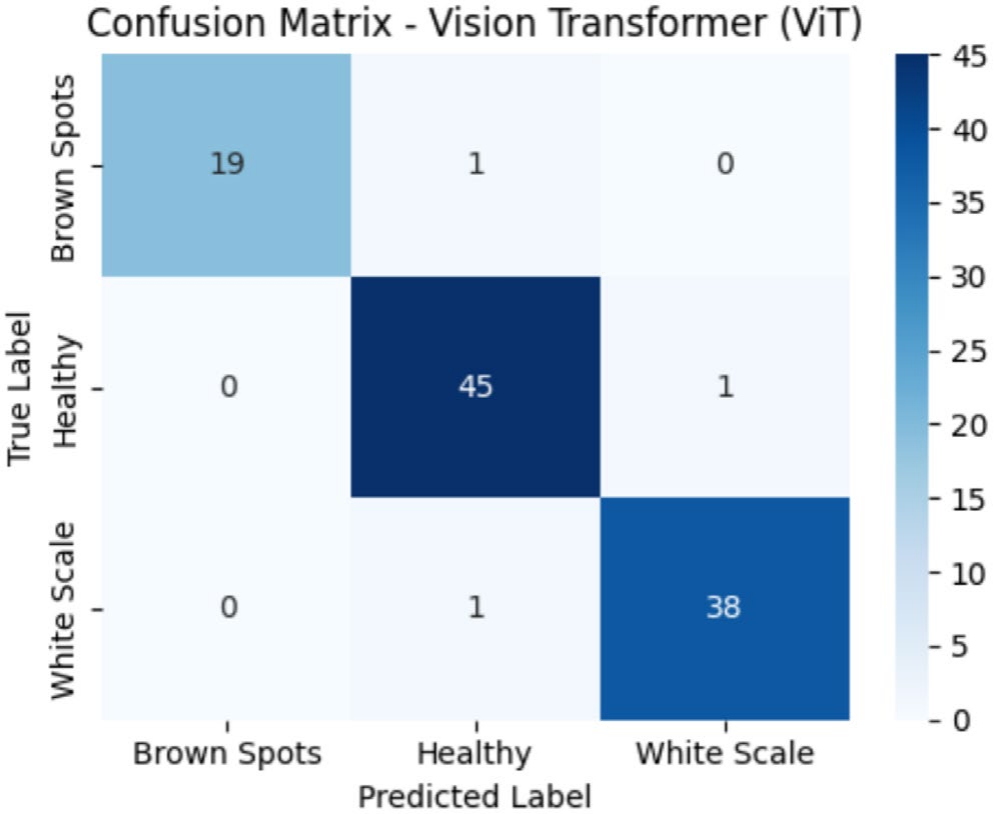
Confusion matrix for ViT model on test data.

Figure 13 demonstrates the confusion matrix of the ViT model, presenting the classification performance concerning the three classes, including Brown Spots, Healthy, and White Scale. The model correctly labeled 19 Brown Spot samples, but wrongly labeled 1 sample as Healthy. It has given good Healthy scores with 45 of 46 correctly called and only one sample wrongly called as White Scale. In the case of White Scale, 38 samples were correctly identified and classified through the model, but one sample was assigned to the wrong category and marked as having Healthy. On balance, the ViT model has high classification accuracy, and the behavior is consistent in all classes. The confusion matrix indicates low misclassification rates, suggesting the model has effectively learned to distinguish between disease classes with high reliability. A slight disorientation, particularly with the Healthy group, could be associated with the faint similarities of the features of early‐stage infections. When compared to the HybridConv Mixer model, the ViT model had fewer misclassifications and higher overall precision, indicating that it was better at recognizing the global patterns relevant to disease identification.

Table 9 shows the classification report of the model ViT that describes the model's performance by classifying the three target classes: Brown Spots, Healthy, and White Scale. In the Brown Spots class, the model achieved a precision of 1.00, indicating that all predicted Brown Spots samples were true. Additionally, it had a recall of 0.95, meaning that 95% of the actual Brown Spots were correctly predicted. This gave an F1‐score of 0.97. The Healthy variant demonstrated even results of precision 0.96 and recall 0.98, resulting in an F1‐score of 0.97. In the case of White Scale, both precision and recall were 0.97 and consequently provided a consistent F1‐score of 0.97. Macro average precision, recall, and F1‐score are all at around 0.97 to 0.98, representing balanced performance of the model in all the classes. The model accuracy is 97%, which implies that most of the predictions made in the test set were correct. Such findings indicate that the ViT model offers a consistent and balanced response to various categories of leaf conditions.

**TABLE 9:** Classification report for vision transformer.

Class	Precision	Recall	F1‐score	Support	Accuracy
Brown spots	1.00	0.95	0.97	20	0.97
Healthy	0.96	0.98	0.97	46	
White scale	0.97	0.97	0.97	39	
Macro avg	0.98	0.97	0.97	105	

### 4.3 Ensemble Model Performance Metrics: Epoch‐By‐Epoch Accuracy and Loss

The ensemble model achieves performance enhancements across 100 epochs based on the data presented in Table 10 through its epoch‐based accuracy and loss evaluation. The table shows that training and validation accuracy continue to rise while loss values drop, consequently showing that the ensemble model successfully classifies data. During epoch one, the training accuracy begins at 23.88% but exhibits a training loss of 2.3417, while validation accuracy measures 49.74% with a value of 1.3188 for validation loss. The model makes significant errors during its initial learning stages, indicating its current state. The model demonstrates rapid progress in epoch 10, with training accuracy reaching 86.37% and validation accuracy attaining 92.17%. Concurrently, loss values decrease significantly, indicating successful feature extraction and learning. At epoch 20, training accuracy reaches 95.31%, while validation accuracy stands at 96.39%, along with validation loss producing a value of 0.1002, which indicates superior classification capability. The model continues to improve its learning during epoch 30, reaching 97.69% training accuracy and 97.18% validation accuracy, which means steady growth. At epoch 40, training accuracy achieves 98.59%, resulting in improved validation accuracy that reaches 98.22%, while loss values keep declining. The model maintains its high performance level after epoch 50 since training accuracy exceeds 99% while validation accuracy stabilizes near 98%. The model effectively learns the dataset when training accuracy reaches 99.73% and validation accuracy sustains 98.22% during epoch 100, while showing minimal loss. The ensemble model demonstrates its strength through combined model operation, which improves learning efficiency and yields high accuracy rates and minimal errors, making it fit for real‐world usage.

**TABLE 10:** Epoch‐wise accuracy and loss for ensemble model.

Epoch	Train accuracy	Train loss	Val accuracy	Val loss
1	0.2388	2.3417	0.4974	1.3188
10	0.8637	0.1297	0.9217	0.2242
20	0.9531	0.1297	0.9639	0.1002
30	0.9769	0.0645	0.9718	0.0813
40	0.9859	0.0387	0.9822	0.0609
50	0.9935	0.0198	0.9817	0.0673
60	0.9934	0.0208	0.9809	0.0713
70	0.9949	0.0162	0.9773	0.1023
80	0.9952	0.0155	0.9819	0.0672
90	0.9943	0.0170	0.9854	0.0582
100	0.9973	0.0090	0.9822	0.0797

**FIGURE 14:**
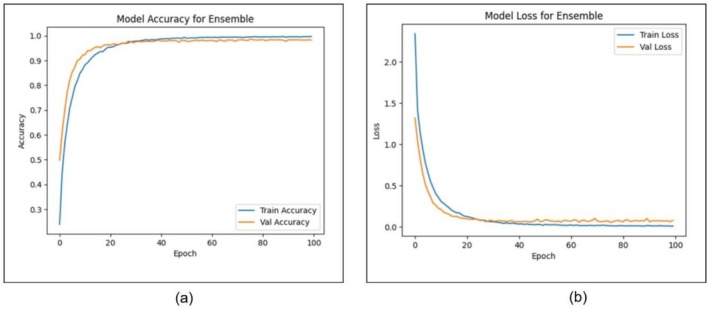
Training and validation results for ensemble model: (a) accuracy and (b) loss.

Figure 14 shows the performance (training and validation) curves of the proposed Ensemble Model according to 100 epochs with two important metrics of accuracy and loss. Subfigures (a) and (b) show how the accuracy of the training and validation changed over time, and the training and validation loss results, respectively. In Figure 14a, one can see that the training and validation accuracy are increasing as the number of epochs increases. The training accuracy rises rapidly in the initial epochs. It stabilizes at over 99%, while the validation accuracy follows a similar trajectory, reaching a nearly identical point at slightly less than 99%. The fact that the two curves resemble each other and have a slight difference is considered to be evidence of the model's good generalization potential. It proves that the overfitting issue has been efficiently addressed. On the same note, the loss curves in Figure 14b further indicate stability and convergence of the model. The training and validation losses drop rapidly in the first few stages, which demonstrates proper learning. The curves start to straighten after around 30 epochs and approach zero, which implies that the model has found a good solution. The validation loss is also quite similar to the training loss, indicating that the model is well balanced in handling both visible and unseen data. These findings affirm the Ensemble Model's stable and strong learning behavior, which shows high predictive accuracy results and minimal loss. The high correlation between the training and validation measures highlights the model's ability to generalize effectively across heterogeneous samples, making it a suitable option for plant disease classification.

#### 4.3.1 Evaluation of Testing Results

**FIGURE 15:**
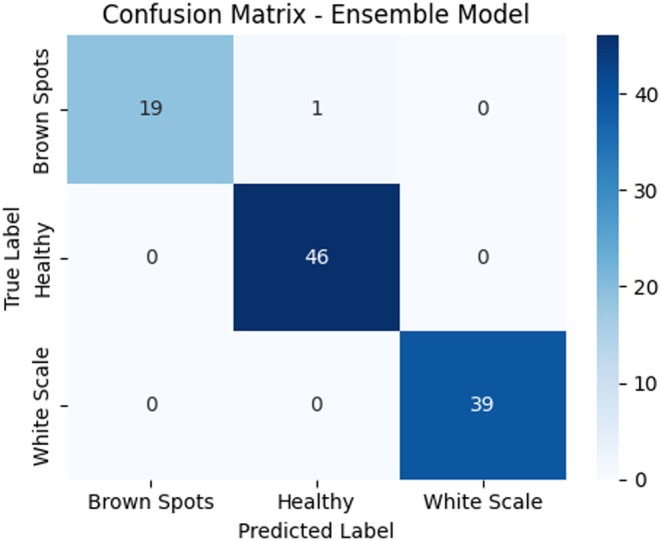
Confusion matrix for HybridConv mixer model on test data.

Figure 15 presents the Ensemble Model confusion matrix, which integrates the outputs of the HybridConv Mixer and Vision Transformer to categorize date palm leaf images into three categories: Brown Spots, Healthy, and White Scale. The classification box shows that 19 (= 95.0%) of Brown Spot samples were correctly classified, and only one sample was classified as Healthy. There were no cases of misclassification, and all 46 samples of Healthy were classified as such, and similarly, all 39 White Scale samples were also classified accurately. As shown in this confusion matrix, the Ensemble Model performs very well, achieving almost perfect results in classification, despite the number of classes. It has better consistency and fewer mistakes compared to the single models, especially when it comes to not confusing visually close classes. The zero misclassification on the Healthy and White Scale and the insignificant error on the Brown Spot class demonstrate the effectiveness of the ensemble strategy in utilizing both local and global characteristics to detect the disease accurately.

The Ensemble Model demonstrates its classification excellence through accuracy and loss measurements. The Ensemble Model classification report in Table 11 summarizes the model's results across the three target classes: Brown Spots, Healthy, and White Scale. In the case of the Brown Spots class, the model had a precision of 1.00, which means that all of the predicted Brown Spot cases were correct, and a recall of 0.95, signifying that it has correctly labeled 95% of real Brown Spot cases where recall is defined as the proportion of actual labeled cases that are correctly identified. This gave an F1‐score of 0.97. In the case of the Healthy class, a precision of 0.98 and an absolute recall of 1.00 were observed, which implies the classification of all Healthy samples fairly, with an F1‐score of 0.99. In the White Scale, all three metrics were perfectly scored using the model, resulting in precision, recall, and F1‐scores equating to 1.00. The macro average precision, recall, and F1‐score were 0.99, 0.98, and 0.99, which is consistent and high performance on all classes. The model's general accuracy level is 99%, indicating that almost all estimations on the test set were accurate. These findings affirm that the Ensemble Model is highly performing, with balanced performance in classifying the conditions of date palm leaves.

**TABLE 11:** Classification report for ensemble model.

Class	Precision	Recall	F1‐score	Support	Accuracy
Brown spots	1.00	0.95	0.97	20	0.99
Healthy	0.98	1.00	0.99	46	
White scale	1.00	1.00	1.00	39	
Macro avg	0.99	0.98	0.99	105	

### 4.4 Comparison of Models

The Ensemble Model exhibits superior classification performance, combining the predictive merits of the Hybrid Conv Mixer and the ViT models. It displays the best total accuracy of 0.99 as shown in Table 12, which implies that it can classify correctly almost all of the input samples. Moreover, it produces macro‐averaged precision, recall, and F1‐score of 0.99, 0.98, and 0.99, respectively, which demonstrates a balanced and more general performance over all the target classes. This indicates that the Ensemble Model not only reduces false positives (high precision) but also captures a larger number of true cases (high recall), making it the most reliable model to use in practice. The advantage of the ensemble method lies in the fact that the constituent models assume low correlation. Although the Conv Mixer might be beneficial in terms of local spatial features capture and the ViT is trying to obtain global attention‐based patterns, the ensemble is also a good combination of both angles. This synergy enables the model to generalize more effectively and to avoid the tendency of overfitting or underfitting in separate architectures. The Ensemble Model also demonstrates the improvement in the quality of the predictions as compared to the standalone models in all the disease categories. Thus, an ensemble approach can also be higher in accuracy than its single counterparts, thereby decreasing classification susceptibility to a greater extent. This makes it a better option in classification terrain where accuracy is needed the most in plant disease diagnostics.

**TABLE 12:** Comparison of classification performance across models.

Model	Precision (avg)	Recall (avg)	F1‐score (avg)	Accuracy
Hybrid Conv Mixer	0.93	0.93	0.93	0.94
ViT	0.98	0.97	0.97	0.97
Ensemble model	0.99	0.98	0.99	0.99

### 4.5 Visual Explanation Using Grad‐CAM

**FIGURE 16:**
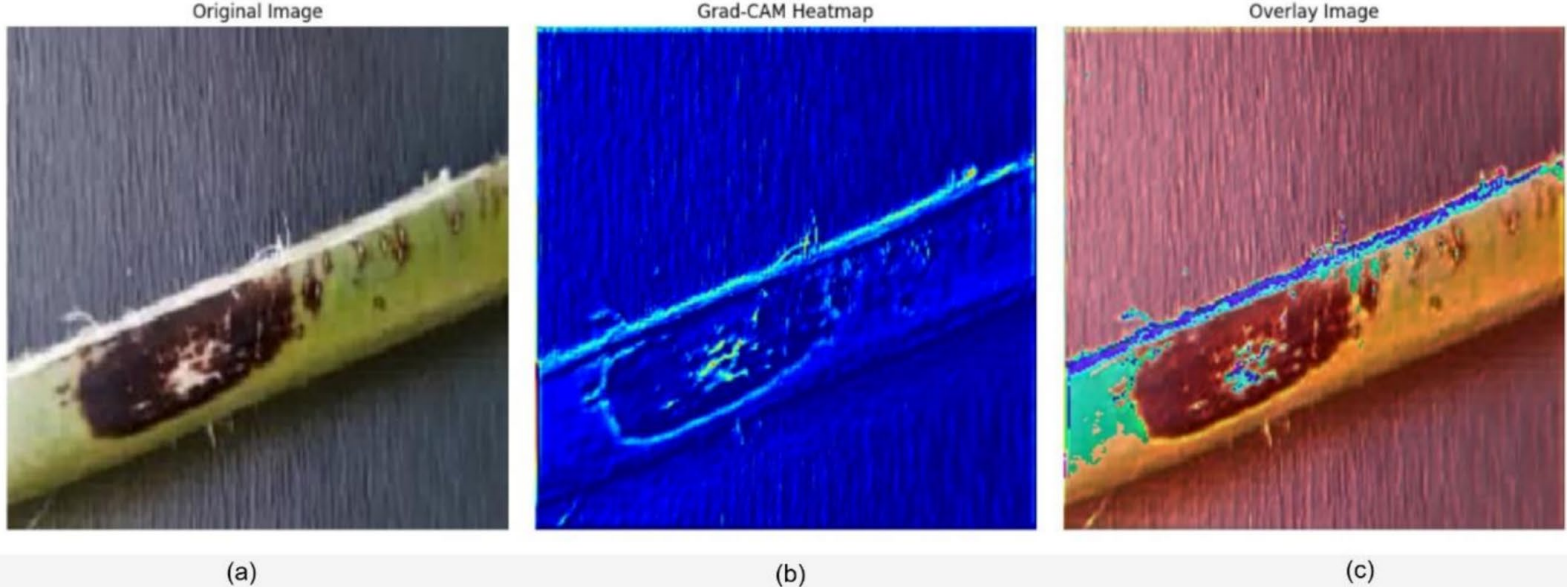
Grad‐CAM‐based interpretability of the ensemble model on a brown spot‐infected date palm leaf. (a) Original input image, (b) Grad‐CAM heatmap showing activation intensity, and (c) overlay of heatmap on the input image highlighting model attention.

To make the predictions of the ensemble model, Gradient‐weighted Class Activation Mapping (Grad‐CAM) was used on a correctly predicted test sample of a date palm leaf with affected brown spots. Figure 16 displays the heatmap of the first panel, Grad‐CAM; the second panel shows the heatmap overlaid on the original input image, and the third panel is the input image. Grad‐CAM provides a visualization of the regions of the leaf that the model focused on when making its prediction. These areas correlate with the declared brown lesions, which are the symptoms of that disease class. The heatmap, colored in blues, effectively highlights the response to infected regions, and the overlay verifies that its concentration is limited to disease‐sensitive areas rather than background sources. This visual elucidation elucidates that the model is not making use of spurious correlations but is focused on biologically meaningful characteristics instead. Grad‐CAM added interpretability to the system, which increases the level of transparency and trust in its final output, which is crucial in agricultural scenarios where the explanation of decisions must be provided to field specialists and farmers.

**FIGURE 17:**
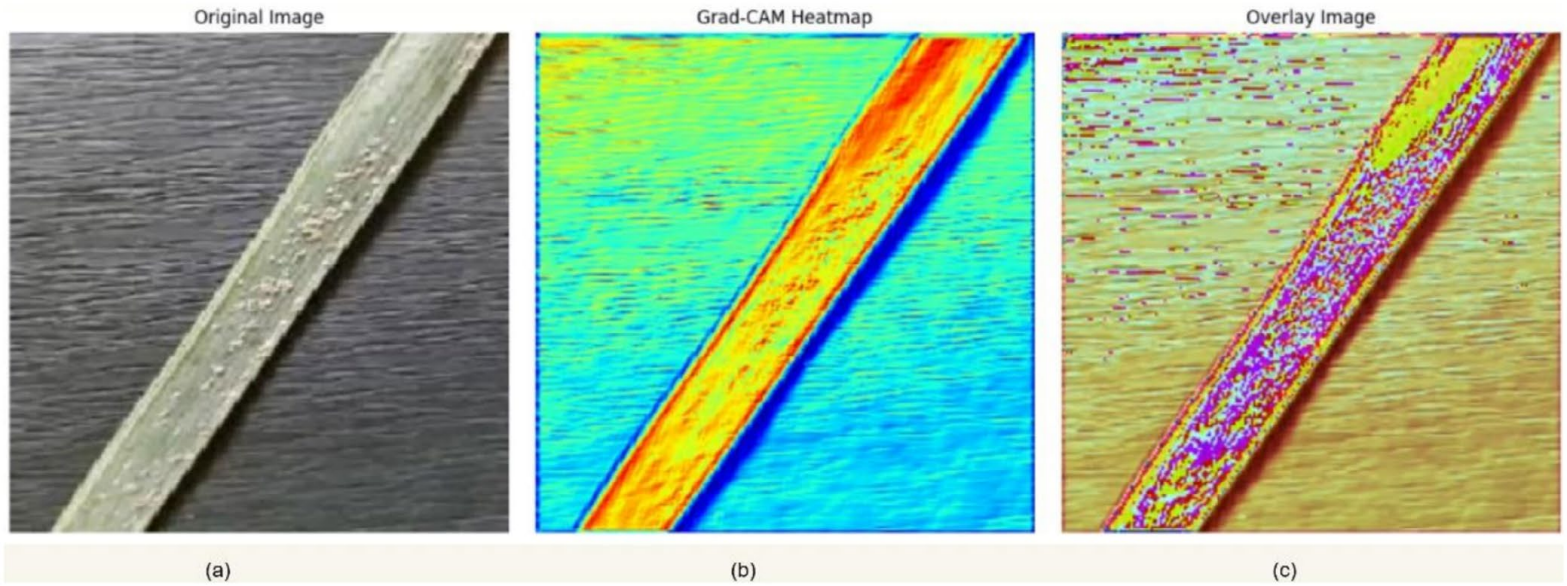
Grad‐CAM‐based interpretability of the ensemble model on a White scale date palm leaf. (a) Original input image, (b) Grad‐CAM heatmap showing activation intensity, and (c) Overlay of heatmap on the input image, highlighting model attention.

Grad‐CAM was then applied to a correctly identified test sample of the White Scale category, allowing for a visual demonstration of how the model made its decision and which image areas were used most significantly. As presented in Figure 17, the visualization has three views: the original image (a), the corresponding Grad‐CAM heatmap (b), and the superposition of the heatmap on the input image (c). This interpretation approach suggests that the model concentrates its attention primarily on the central leaf area, where symptoms of the white scale are most evident. The heatmap intensity of such regions proves that the infected regions were the factor that contributed to the prediction and not background noise. This narrow type of attention clearly highlights the model's competency in identifying salient visual markers related to the disease. This aids in developing confidence in using it to perform actual‐world diagnostic activities; consequently, judgments are made based on facts of discovery of actual pathology on the palm leaf, rather than inarticulate anomalies.

### 4.6 Misclassification

**FIGURE 18:**
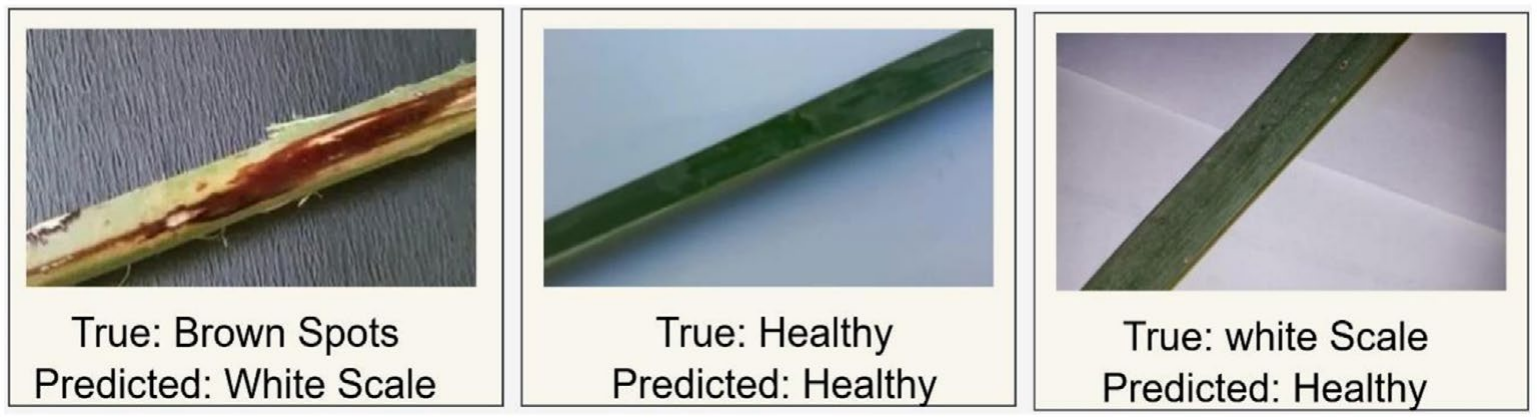
Representative misclassifications on test images.

During automated classification of date palm diseases, similar appearances of various diseases result in problematic misclassification. To illustrate such confusions, several classification results adopted in Figure 18 depict correct and incorrect predictions. In one of the samples, a Brown Spot‐infected leaf was misclassified as White Scale, likely due to the similarity in lesion patterns with those caused by wax systems, such as scale infestation. The second picture presents a Healthy date palm leaf that has been properly assigned and proves that the model recognizes the non‐diseased leaves when recognizable visual features like consistent texture and coloring are found. Conversely, a third picture involves a White Scale‐affected leaf that was erroneously identified as healthy, possibly because faint or initial‐stage symptoms are not visually conspicuous. These demonstrations show how easily classification can be misled simply by using visual patterns, and it is crucial to have adequate feature extraction methods. They also mention the necessity of running models on carefully balanced and diverse data to allow them to differentiate between minor nuances of the disease. These types of misclassifications help us learn the behavior of the models and inform any future updates in practical agricultural disease detection methods.

### 4.7 Ablation Study on Model Components

The ablation of study has been performed to evaluate the individual performance of the HybridConv Mixer and Vision Transformer to the overall performance. The independent evaluation was performed on each component with the same conditions of experiments, and the outcomes were compared to the suggested HybridConv + ViT ensemble. Table 13 illustrates that HybridConv Mixer was the only one that reached the accuracy of 94% and ViT was more accurate with 97%. The suggested ensemble performed better than either, having 99% accuracy with steadily greater precision, recall and F1‐scores. These findings support the rationale behind our hybrid architecture: HybridConv works well at local spatial features (the textures of leaves and lesions), whereas ViT models long‐range correlation and global contextual information. The combination of their strengths is complementary, and the classification performance is much better than in either of the two models.

**TABLE 13:** Ablation study on Kaggle Date Palm dataset.

Model	Precision (avg)	Recall (avg)	F1‐score (avg)	Accuracy
HybridConv Mixer	0.93	0.93	0.93	0.94
Vision Transformer	0.98	0.97	0.97	0.97
HybridConv + ViT (Proposed)	0.99	0.98	0.99	0.99

### 4.8 Computational Efficiency Analysis

As the proposed model is meant to be applied in practice to agriculture, with a possibility of its implementation on edge devices, like drones or mobile phones, we compared its computational efficiency to standard baselines. Table 14 presents the comparison in the parameter count, FLOPs, model size and inference speed. Classical CNN models (MobileNet, Xception) had reduced footprints but worse accuracy (9296%). Models based on transformers (ResNet 50 and AlexNet) were found to be less accurate (approximately 97%) but had much higher numbers of parameters and higher FLOPs. The HybridConv + ViT Ensemble was the best performing, with an overall accuracy of 99%, a moderate complexity of 42 M parameters, 9.8 GFLOPs and approximately 42 ms per image inference time. These findings indicate a good ratio between efficiency and foresight, which proves that the model can be realistically applied. Also, pruning, quantization, and lightweight backbone replacement are optimization methods that can be used in future efforts to remain even more computationally lightweight in edge deployment.

**TABLE 14:** Computational efficiency comparison of models on Kaggle Date Palm dataset.

Model	Parameters (M)	FLOPs (G)	Model size (MB)	Inference speed (ms/image)	Accuracy
MobileNet	5.2	1.1	21	18	92%
Xception	138.3	15.5	528	65	95%
ResNet50	25.6	4.1	98	29	96%
Alex Net	88.0	15.2	345	69	97.5%
HybridConv + ViT (proposed)	42.1	9.8	165	42	99%

Although the raw accuracy numbers suggested that the proposed HybridConv + ViT Ensemble was better that HybridConv and ViT, we further performed statistical significance test to verify that the gains were not due to random error. Paired classification outputs were compared using McNemar test and bootstrap resampling procedure (10,000 times) was used to estimate accuracy differences with 95% confidence intervals. As detailed in Table 15, Ensemble increased its accuracy by 5.0% compared to HybridConv (95% CI: [4.1%, 5.6%], *p* < 0.001) and 2.0% compared to ViT (95% CI: [1.5%, 2.3%], *p* < 0.01). Even ViT was much better than HybridConv by a margin of 3.0% (95% CI: [2.5%, 3.4%], *p* < 0.01). The findings made by these results show that the performance of the Ensemble is not only statistically significant but also not by chance, and so it is highly justified to consider the hybrid architecture as proposed.

**TABLE 15:** Statistical significance analysis of model performance.

Comparison	Accuracy difference	95% CI (bootstrap)	McNemar's test (*p*)	Significance
Ensemble vs. HybridConv	+5.0%	[4.1%, 5.6%]	< 0.001	Yes (highly significant)
Ensemble vs. ViT	+2.0%	[1.5%, 2.3%]	< 0.01	Yes (significant)
ViT vs. HybridConv	+3.0%	[2.5%, 3.4%]	< 0.01	Yes (significant)

### 4.9 Additional Evaluation on External Dataset

In order to further test the possibility of generalization of the proposed HybridConv Mixer + Vision Transformer ensemble model outside of the 1st Input dataset, we conducted an independent test with the use of the publicly available dataset Infected Date Palm Leaves by Dubas Insects (Hamaidi 2025). This data varies in terms of the types of diseases, conditions of acquisition, and appearance; therefore, it serves as a realistic benchmark on external validation. The overall accuracy of the model was 98%, and the class‐specific precision, recall, and F1‐scores were mostly within the range of 0.97–0.99. Precisely, it was found that Healthy leaves were identified with 0.97 accuracy and 0.98 recall, whereas classes related to the infection (Bugs only, Honeydew, and Mixed) had F1‐scores of 0.97 to 0.98. The results summarized in Table 16 indicate that the model maintains high performance when it is tested on external data that is not known; thus, it proves that the model is robust enough but significantly lowers the chances of the 99% accuracy that was achieved on the Kaggle data being an overfitting. This cross‐dataset test supports the assertion that the given framework can be effectively generalized to real‐life situations associated with the detection of date palm disease.

**TABLE 16:** Classification results of proposed model on second dataset.

Class	Precision	Recall	F1‐score	Support	Accuracy
Healthy	0.97	0.98	0.98	200	0.98
Infected—Bugs	0.98	0.97	0.97	150	
Infected—Honeydew	0.99	0.98	0.98	200	
Infected—Mixed (Bugs + Honeydew)	0.98	0.99	0.98	200	
Macro Avg	0.98	0.98	0.98	750	

### 4.10 Benchmarking Against Standard Architectures

To provide a well‐balanced methodologically valid comparison, we have compared the proposed model HybridConv Mixer + ViT Ensemble model to several of the popular deep learning models, namely MobileNet (Pacal and Işık 2025), Xception (Safran et al. 2024), ResNet50 (Namoun et al. 2024), and AlexNet (Alshehhi et al. 2022). It is compared in terms of average precision, recall, F1‐score, and accuracy, and the results are summarized in Table 17. MobileNet was found to have the lowest accuracy of 92 and a precision and recall of 0.91–0.92, indicating its water‐efficient architecture instead of its maximum‐accuracy architecture. Xception and ResNet50 were even more successful with their 95 and 96% accuracy, respectively, and equal precision and recall. Though AlexNet is a more basic CNN architecture, it was used in the classification task with 97.5% accuracy, which demonstrates its usefulness in the current classification problem. On the contrary, the presented HybridConv + ViT Ensemble model was significantly better than the baselines (99% accuracy) and also had a higher preciseness (0.99), recall (0.98), and F1‐score (0.99). This performance justifies the subsequent idea of our hybrid design: convolutional layers in HybridConv pick fine‐grained local texture lesions and scale deposits, whereas the Vision Transformer is effective in modeling long‐range dependencies and global contextual information. The combination of them gives the advantage of complementary strengths, which leads to the more powerful and generalizable plant disease detection framework. These results show that the suggested model operates better than both CNN‐based and transformer‐based baselines, which proves the appropriateness of its application to the real world of agriculture.

**TABLE 17:** Comparison of standard models and the proposed ensemble on the Kaggle Date Palm dataset.

References	Model	Precision (avg)	Recall (avg)	F1‐score (avg)	Accuracy
Pacal and Işık (2025)	MobileNet	0.91	0.92	0.91	0.92
Safran et al. (2024)	Xception	0.94	0.95	0.94	0.95
Namoun et al. (2024)	ResNet50	0.95	0.96	0.95	0.96
Alshehhi et al. (2022)	Alex Net	0.97	0.97	0.97	0.975
Proposed Ensemble Model		0.99	0.98	0.99	0.99

## 5 State of the Art Comparison

Table 18 provides a comparative discussion of some of the state‐of‐the‐art studies that are available and performed on date palm disease categorization using various data sets and deep learning techniques. Alaa et al. (2020) obtained results of 97.9% and 92.8% accuracy with CNN and SVM, respectively. Alshehhi et al. (2022) formulated a dataset of leaf discoloration and implemented transfer learning models, such as SqueezeNet, GoogleNet, and AlexNet, and achieved 98% accuracy. Al‐Mulla et al. (2023) utilized aerial ORS data collected via drones and high‐resolution remote sensing technologies to detect parasitic infestation by the Dubas bug, achieving 87% accuracy. Magsi et al. (2023) specialized in Sudden Decline Syndrome using their collected dataset and MobileNet, achieving an accuracy of 60%. Hessane et al. (2023b) employed a fuzzy clustering strategy on a dataset of a white‐scale disease, achieving a classification accuracy of 94.18%. By comparison, the proposed work utilizes a Kaggle dataset comprising three classes of diseases. It incorporates a Hybrid Conv Mixer and ViT into an ensemble architecture, outperforming others with a reported accuracy of 99%, thereby confirming the efficacy of integrating local and global features in disease detection.

**TABLE 18:** Comparative analysis of state‐of‐the‐art method.

References	Year	Authors	Dataset used	Approach	Reported accuracy
Namoun et al. (2024)	2024	Namoun et al.	Public Kaggle dataset: “Infected vs Healthy Date Palm Leaves”	ResNet50, EfficientNet, MobileNet V2	96% EfficientNet
Alaa et al. (2020)	2020	Alaa et al.	Custom dataset	CNN and SVM	97.9% and 92.8%
Alshehhi et al. (2022)	2022	Alshehhi et al.	Custom leaf discoloration dataset	SqueezeNet, GoogleNet, and AlexNet	98%.
Al‐Mulla et al. (2023)	2023	Al‐Mulla et al.	Drone‐collected aerial images (Wadi Bani Kharus region)	Advanced remote sensing tools	87%
Pacal and Işık (2025)	2023	Magsi et al.	SDS leaf dataset (Sudden Decline Syndrome), custom‐collected	MobileNet	60%
Pacal (2024)	2023	Hessane et al.	Custom white‐scale disease image dataset	Hybrid fuzzy fast multi‐Otsu K‐Means (FFMKO) algorithm	94.18%
Proposed Work			Public Kaggle dataset: Date Palm Data	Hybrid Conv Mixer, ViT, and ensemble model	99%

Numerous studies have explored the potential of artificial intelligence and deep learning in plant disease classification and diagnosis. Bhargava et al. (2024) provided a comprehensive review of AI‐driven plant leaf disease detection using computer vision, emphasizing the relevance of CNNs, transfer learning, and attention‐based models in enhancing classification performance. Similarly, Demilie (2024) presented a comparative study of various classification techniques, highlighting the performance trade‐offs among conventional and deep learning‐based approaches. Tandekar and Dongre (2023) contributed by reviewing image processing methods in plant disease detection, which reinforces the importance of visual features in early diagnosis systems.

Several recent studies offer valuable insights that align with the scope of a Hybrid Deep Learning Model for Date Palm Disease Classification, particularly one that fuses HybridConv Mixer and Vision Transformer architectures. Sharma, Gupta, et al. (2025) proposed the DBA‐DeepLab model, which integrates a dual backbone with attention‐enhanced DeepLab V3+ for precise plant disease segmentation. This architecture effectively captures spatial and semantic details, making it well suited for segmenting infected regions on complex foliage like date palm leaves. Similarly, Singh et al. (2025) presented a U‐Net–based deep learning approach for enhanced leaf disease segmentation. Their model employs multi‐scale contextual learning, which parallels transformer‐based mechanisms and supports accurate disease localization—an essential component for high‐precision classification tasks. Rani et al. (2025) introduced VGG‐EffAttnNet, a hybrid classification model combining VGG16 and EfficientNetB0 with an attention mechanism. This fusion closely mirrors the hybridization of convolutional and transformer features, demonstrating how attention‐guided dual backbones significantly improve classification accuracy and model generalizability. Collectively, these works provide a strong foundation for adapting hybrid attention‐based deep learning models to the domain of date palm disease detection, promoting accurate, interpretable, and scalable solutions for smart agriculture.

Kamilaris and Prenafeta‐Boldú (2018) outlined early contributions of DL models in smart farming, while Hasan et al. (2020) provided an extensive analysis of plant disease classification architectures and datasets. These works reinforce the foundation for deploying hybrid models such as ours in practical agricultural settings.

From a computer vision perspective, Zhang et al. (2025) proposed adaptive keypoint extraction across diverse scenes, which is valuable for understanding complex leaf textures. Wang et al. (2025) applied an improved YOLOv8 in underwater detection, highlighting the importance of efficiency in challenging environments, paralleling our model's goal of effective disease classification under varied leaf conditions. In the specific context of date palm classification, Safran et al. (2024) introduced DPXception, a lightweight CNN tailored for date palm species, which supports the feasibility of specialized models for date palm datasets. Nasra et al. (2025) and Sharma, Al‐Huqail, et al. (2025) further support the use of CNN‐based architectures and feature extraction techniques, confirming the significance of leveraging ResNet50 and MobileNetV2 architectures approaches in plant disease recognition systems.

## 6 Conclusion

The paper demonstrates the effectiveness of deep learning models in categorizing Brown Spots and White Scale diseases of date palm leaves from visual images. The three architectures that were considered include the HybridConv Mixer, ViT, and an ensemble model that combines both. The ensemble model performed the best in terms of classification accuracy (99%) compared to both ViT (97%) and HybridConv (94%). The combination of locally based textual features of CNN layers with global context features of transformers enabled effective disease detection. To improve the interpretability of the forecast, Grad‐CAM visualizations were performed on correctly classified examples of samples from various disease classes, ensuring the model focuses on biologically significant areas of the leaf. Such visual descriptions enhance confidence in the decisions made by the model and aid in the possible applications of the model in the real practice of precision agriculture.

## 7 Future Directions

To apply the model to larger, more heterogeneous data sets in the future, we will inferently work on extending the model to both larger and more novel environmental and imaging conditions with the help of domain adaptation methods to address distributional variations. Pruning, quantization, and lightweight backbones (e.g., EfficientNetV2, MobileViT) as options in computational optimization will also be considered to make effective use of edge computing devices such as drones and smartphones to support real‐time monitoring in the field. We also expect to scale the framework and forecast the severity and level of disease progression, combine new explainable AI methods, and compare the model with the latest architectures like DeiT and ConvNeXt, which will further enhance the scalability and usability of the architecture in precision agriculture.

## Author Contributions

**Taifa Ayoub Mir:** conceptualization (equal), methodology (equal), software (equal), writing – original draft (equal). **Salil Bharany:** data curation (equal), formal analysis (equal), investigation (equal), visualization (equal). **Rupesh Gupta:** data curation (equal), validation (equal), writing – review and editing (equal). **Rania M. Ghoniem:** formal analysis (equal), project administration (equal), resources (equal), writing – review and editing (equal). **Ateeq Ur Rehman:** conceptualization (equal), methodology (equal), project administration (equal), writing – review and editing (equal). **Belayneh Matebie Taye:** formal analysis (equal), investigation (equal), project administration (equal).

## Ethics Statement

No animals or human subjects were involved in this study. The study utilized publicly available datasets, and all methods were carried out in accordance with relevant guidelines and regulations.

## Conflicts of Interest

The authors declare no conflicts of interest.

## Data Availability

The dataset used in this study is publicly available on Kaggle: “Date Palm Data,” Kaggle Datasets, March 2025. [Online]. Available: https://www.kaggle.com/datasets/hadjerhamaidi/date‐palm‐data?resource=download. Accessed: March 5, 2025.
